# Target templates and the time course of distractor location learning

**DOI:** 10.1038/s41598-022-25816-9

**Published:** 2023-01-30

**Authors:** Aylin A. Hanne, Jan Tünnermann, Anna Schubö

**Affiliations:** Cognitive Neuroscience of Perception and Action, Department of Psychology, Philipps-University Marburg, Marburg, Germany

**Keywords:** Attention, Psychology

## Abstract

When searching for a shape target, colour distractors typically capture our attention. Capture is smaller when observers search for a fixed target that allows for a feature-specific target template compared to a varying shape singleton target. Capture is also reduced when observers learn to predict the likely distractor location. We investigated how the precision of the target template modulates distractor location learning in an additional singleton search task. As observers are less prone to capture with a feature-specific target, we assumed that distractor location learning is less beneficial and therefore less pronounced than with a mixed-feature target. Hierarchical Bayesian parameter estimation was used to fit fine-grained distractor location learning curves. A model-based analysis of the time course of distractor location learning revealed an effect on the asymptotic performance level: when searching for a fixed-feature target, the asymptotic distractor cost indicated smaller distractor interference than with a mixed-feature target. Although interference was reduced for distractors at the high-probability location in both tasks, asymptotic distractor suppression was less pronounced with fixed-feature compared to mixed-feature targets. We conclude that with a more precise target template less distractor location learning is required, likely because the distractor dimension is down-weighted and its salience signal reduced.

## Introduction

Our visual environment is exceedingly manifold holding a tremendous amount of constantly changing information. Since the processing resources of the visual system are limited, humans need to prioritize relevant information over irrelevant information by using visual selective attention. Usually, the prioritization of relevant information works very well ensuring an efficient, target-aimed selection of visual information^1,2^. However, occasionally, visually salient information that is not relevant to current goals involuntarily captures our attention and interferes with our search for a target^3,4^.

Studies examining attentional control often use the additional singleton search task^5,6^. In this task, observers are asked to search for a shape singleton presented among a set of non-targets (e.g. a diamond among circles or vice versa). In some trials, one of these non-targets is displayed in a different colour, becoming a distracting colour singleton. The presence of the colour distractor interferes with the search for the target, which manifests in increased response times (distractor presence cost)^7^. Distractor interference occurs even though the colour distractor is irrelevant to the task and the shape singleton target and the colour distractor are defined on different feature dimensions. Based on these findings, Theeuwes^8^ proposed that the first shift of spatial attention is determined by the most salient object within the visual field, independent of the observer’s internal representation of the target^3,8^. However, Bacon and Egeth^9^ modified the additional singleton search task by presenting non-targets with different shapes (e.g. triangles, squares, diamonds) and asking observers to search for a target with a fixed feature (e.g. a circle). With this manipulation, observers could no longer search for a shape singleton, but had to set up a precise, namely a feature-specific target template. No distractor interference was found, indicating that searching for a particular target feature (feature search) had reduced the sensitivity for attentional capture. In contrast, in tasks where the features of target and distractor change unpredictably across trials^5,10–14^ observers cannot search for a fixed target feature^15^ but have to adopt a singleton-detection mode^9^ to find the target. Interestingly, and even though singleton-detection mode makes observers more susceptible to attention being captured by the distractor, it seems that many observers prefer this suboptimal search strategy when not prevented by further instructions^16–18^. Although there is a trend to choose the easiest search mode available (e.g., Irons and Leber^16^; see also Theeuwes^8^), participants also tend to stick with the search mode they experienced as being effective in previous search trials (e.g., Leber and Egeth^19^).

Other studies have also shown that a fixed-feature target, as opposed to a mixed-feature target, allows observers to down-weight a feature dimension that is not task-relevant^10,12^. Also, the dimension-weighting account suggested that signals are weighted dependent on their relevance for a task on a dimensional level. Whereas processing of signals from up-weighted feature dimensions is facilitated, signals from non-relevant dimensions are attenuated^20–23^. Thus, in tasks with a target defined in a dimension that does not overlap with the dimension of the distractor, observers can up-weight the task-relevant and down-weight the task-irrelevant dimension to protect the target against a more salient distractor.

Studies investigating distractor location learning used a variant of the additional singleton search task^12,13,24–27^, in which a colour distractor was more likely to appear at one of the display locations. Two main results were reported: First, distractor interference was reduced when the distractor was presented at the high-probability location compared to other display locations. Second, when the target was presented at the high-probability location of the distractor, target selection was impaired (but see Allenmark et al.^28^; van Moorselaar et al.^12^). These findings indicate that the high-probability location was suppressed relative to the other display locations, which resulted in an attenuation of the salience signal of stimuli presented at that location^13,29,30^. Presumably, the weights within the spatial priority map, based on which attention is deployed, were adjusted so that the high-probability location was assigned a low priority to prevent that the distractor captures attention^13,29,30^. Further research demonstrated that distractor location learning is modulated not only by learning of spatial, but also of nonspatial distractor regularities^24,25,31^. Failing et al.^24^ associated two high-probability locations with a particular distractor feature, and found faster response times when the distractor was displayed at its feature-matching high-probability location. This provides evidence that distractor location learning can include featural information as well. In line with this, Stilwell et al.^31^ found feature-based learning, even though pure spatially-based, feature-blind suppression would have been possible as well. In their experiment, the distractor location was 100% predictable with the target never appearing at the distractor location. Feature regularities of the distractor were manipulated by presenting the distractor either in a high- or a low-probability colour. Results showed reduced distractor interference when the distractor appeared in a high-probability colour compared to a low-probability colour, indicating feature-based learning.

In contrast, van Moorselaar et al.^25^ found that a classification algorithm trained on distractor spatial frequencies in pre-stimulus EEG data failed to decode the spatial frequency of the target, supporting the notion of different representations for target and distractor features.

Only a few studies have examined the effects of the target template (i.e., search for a feature-specific target vs. a shape singleton) on distractor location learning^29,32^. Wang and Theeuwes^32^ hypothesized that in feature search mode^9^, distractors can be ignored by top-down processes, eliminating the need for distractor location learning. In their study, observers were asked to search for a unique target shape (circle) among different non-target shapes (diamonds, squares and triangles), similar to the study by Bacon and Egeth^9^. Observers had to search for a fixed feature and could thus use a feature-specific target template to find the target, whereas in other distractor location learning tasks, observers had to search for a shape singleton, which prevented the use of a feature-specific target template^13,24,26^. In contrast to their prediction, they found that a feature-specific target template does not eliminate the need for distractor location learning. It is important to note, however, that the search displays differed substantially between experiments^13,29,32^ making it difficult to directly compare the results. For instance, if the non-target items presented in the search display differ in their similarity, e.g., by having different shapes (e.g. diamonds, squares and triangles), the salience of the singleton distractor might also be reduced^33–35^. Because the salience of a stimulus drives the degree of attentional capture^8,36^, a less salient distractor reduces the need for suppression. Moreover, there is evidence that the target template can be adjusted flexibly to maximize the distinctiveness of target and distractor^15,37^ and that the distractor context can modulate template precision^38^. In a visual search task, Geng et al.^15^ systematically varied the similarity of target and distractor colours in LAB colour space to be at smaller or larger distance to the target, resulting in high similarity of target and distractor features at small distances and low similarity at large distances. After the visual search task, observers were asked to report the target colour on a colour wheel. The results showed a shift of the target template away from the distractor colour. The authors suggested that observers can flexibly adjust their target template to increase the distinctiveness between target and distractor to reduce distractor interference. Thus, the target template is determined by the definition of the target features, but also by the similarity of the non-targets and the similarity between target, non-target and distractor features.

**Figure 1:**
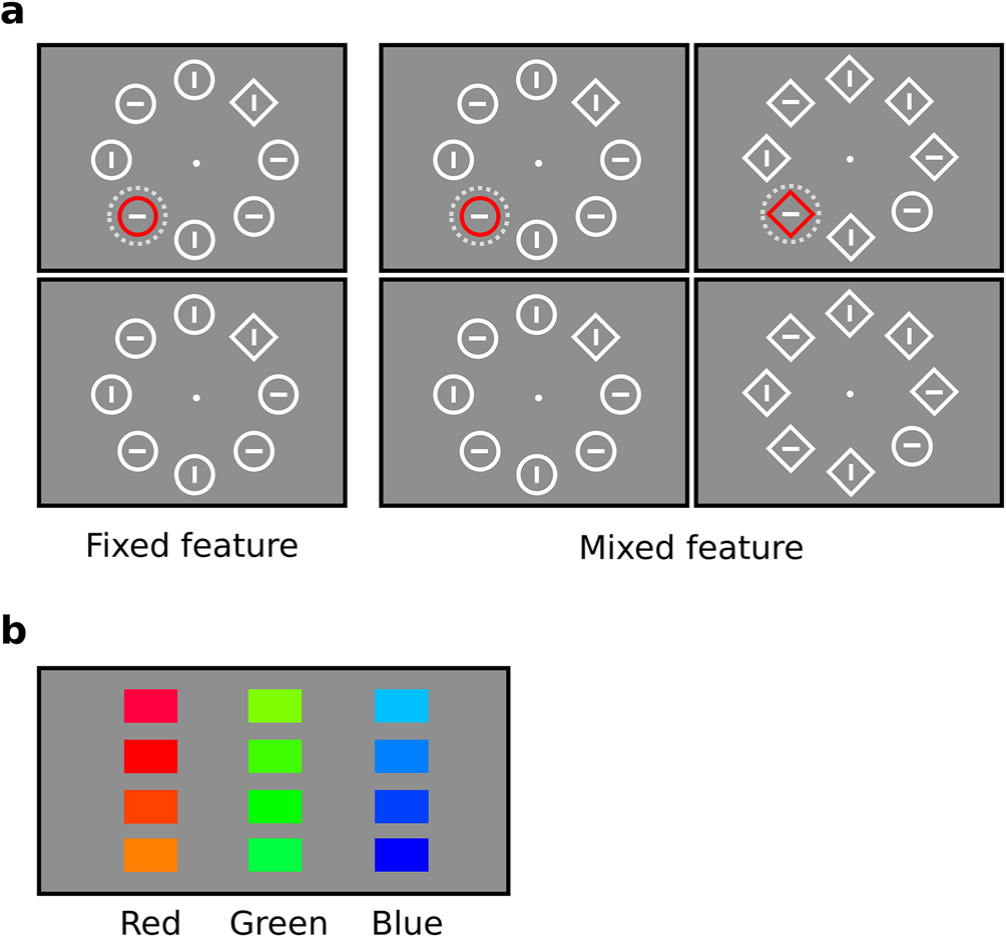
Stimuli and task. (**a**) Examples of the visual search displays. Participants search for a fixed-feature target (diamond; left panel) or a mixed-feature target (diamond or circle; right panels) and report the orientation of the line embedded in it. In 2/3 of the distractor-present trials (top row) the colour distractor was presented at the high-probability location. The high-probability location was chosen randomly for each participant (illustrated by the dotted line, not visible in the experiment). In 50% of trials, no additional colour distractor was presented (bottom row). (**b**) Colour-array. Two out of three colours (red, green, blue) were chosen randomly for each participant. Participants were asked to select a colour hue for each of these two colours. The chosen hues became the colours of the colour distractor.

With this study, we tested whether the target template affects distractor location learning in two variants of the additional singleton search task. Studies interested in varying the search modes participants use (feature search vs. singleton search)^9^, often use arrays with heterogeneous non-targets to prevent that participants use singleton detection mode to find the target^7,14^. We chose a different approach. As the focus of our study was on the precision of the target template rather than on the impact of different search modes, the target features were chosen to allow participants to search with either a precise, feature-specific target template (fixed-feature task); or they had to rely on a much coarser template that only included the information that the target is a singleton in the shape dimension (mixed-feature task). Examples of search arrays are shown in Fig. 1. In the fixed-feature task, the target shape, a diamond, remained constant throughout the experiment; in the mixed-feature task, the target was either a diamond among circles or vice versa. The distractor was coloured randomly in one of two colours, making suppression on the feature level ineffective. Important to note: the non-target and distractor features were kept constant between tasks to not affect salience calculations within the visual field^3^ and to guarantee that performance remained comparable. With this design, we expect less distractor interference in RT in the fixed-feature task because participants can use a feature-specific target template that never overlaps with the distractor features in any of the trials. In the mixed-feature task, which does not allow for a feature-specific target template, target selection should be more prone to attention being captured by the distractor, thus we expect larger interference in RT^12^.

In addition, the spatial distribution of the distractor was biased systematically to assess distractor location learning. We expect that participants learn to suppress a distractor that is presented at the high-probability location, thereby preventing attentional capture of distractors presented at that location compared to distractor presentations at low-probability locations^12,13,24–27^. Moreover, if search with a feature-specific target template allows down-weighting of the distractor feature, there should be less need for distractor location learning in the fixed-feature compared to the mixed-feature task.

Furthermore, as the distractors appeared at locations in varying distance from the high-probability location, the design allowed us to explore how spatial suppression radiates outward from the high-probability location. To that end, we generated a data-driven visualization of the suppression map that illustrates how spatial suppression expands across the visual field.

Distractor location learning is a process that unfolds over time as the observer samples more and more information about the search display, and especially the distractor probabilities in different locations. We assume that the time course with which spatial suppression emerges depends on the precision of the target template and therefore varies between tasks. To reveal such differences, we implemented a learning curve model (see, e.g., Bergmann et al.^39^). The model enables us to assess the *asymptotic distractor cost* that remain after extended experience with the tasks.

Note that the asymptotic distractor cost in the low-probability condition where observers cannot benefit from any location regularity provides an estimate of *distractor interference (DI)* in each task. The difference of the asymptotic distractor cost between the low- and high-probability condition (the *asymptotic suppression effect, SE*) provides us with an estimate of distractor location learning. In line with our reasoning above, we assume *DI* and *SE* to be smaller in the fixed-feature task. Moreover, the model estimates the speed with which these asymptotes are reached. In such a model, the hypothesized differences in capture and distractor location learning could manifest in the asymptotes (*DI*, *SE*), the decrease rates, or in both.

## Results

To examine whether there was less distractor interference and reduced distractor location learning in the fixed-feature task compared to the mixed-feature task, we first performed analyses based on the RTs averaged over the whole time course of the experiments using ANOVAs, a common approach in the field. Second, we conducted a model-based analysis of the spatial gradient of suppression, also using RTs averaged over the time course, to quantify how suppression is distributed across the visual field. Finally, we fitted a model based on learning curves to provide a more fine-grained picture of distractor location learning over time.

### Distractor interference with fixed and mixed features

Response times (RTs) were submitted to a two-way mixed-design ANOVA with the within-participants factor distractor presence (distractor present, distractor absent) and the between-participants factor target (fixed-feature target, mixed-feature target). The latter was implemented as a between participants factor to avoid carryover effects of prior experience on attentional control sets across tasks^19^. We found slower response times in distractor-present trials (*M* = 708.04 ms, *SEM* = 12.38 ms) vs. distractor-absent trials (*M* = 684.82 ms, *SEM* = 11.65 ms) (*F*_(1,58)_ = 67.61, *p* < 0.001, η_p_^2^ = 0.54). Response times were faster in the fixed-feature task (*M* = 639.80 ms, *SEM* = 8.46 ms) compared to the mixed-feature task (*M* = 753.07 ms, *SEM* = 10.64 ms) (*F*_(1,58)_ = 35.80, *p* < 0.001, η_p_^2^ = 0.38)*.* However, the expected interaction effect could not be detected (*F*_(1,58)_ = 1.08, *p* = 0.304, η_p_^2^ = 0.02).

### Distractor location learning

**Figure 2:**
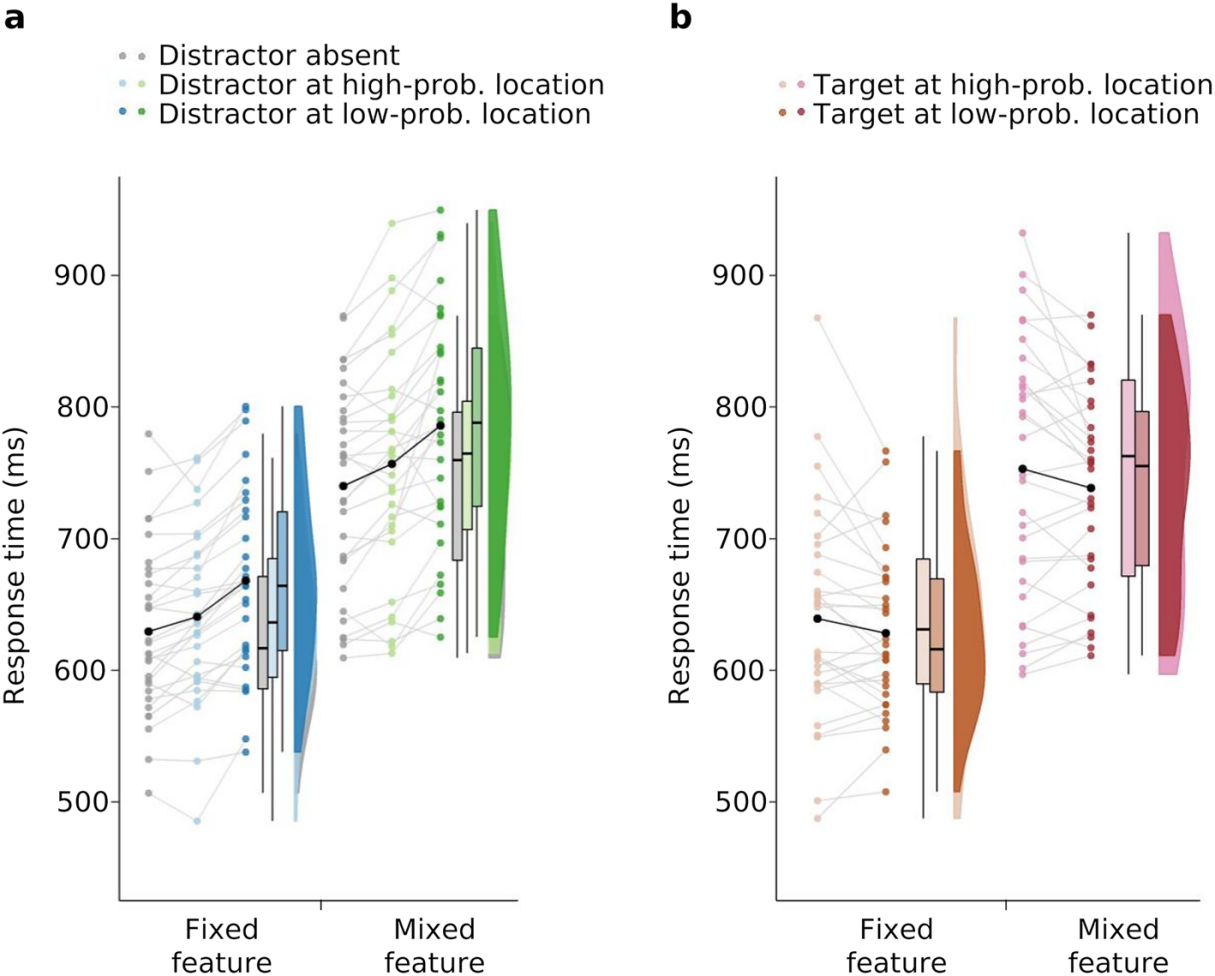
Search response times for fixed-feature targets (left side of the panels) and mixed-feature targets (right side of the panels). (**a**) Box-whisker plots show mean response times as a function of distractor-absent trials (grey), and distractor-present trials (coloured), with the distractor presented at the high-probability location (light blue and green) and at the low-probability locations (dark blue and green). (**b**) Box-whisker plots show mean response times in distractor-absent trials (coloured), with the target presented at the high-probability distractor location (light orange and pink) and at the low-probability distractor locations (dark orange and pink). Coloured and grey dots show response times for individual participants. Mean response times are shown in black.

Suppression of the high-probability location was examined by submitting mean RTs to a two-way mixed-design ANOVA with the within-participants factor distractor location (high-probability, low-probability, distractor absent) and the between-participants factor target (fixed-feature target, mixed-feature target). The ANOVA showed main effects of both factors (distractor location): *F*_(2,116)_ = 66.60, *p* < 0.001, η_p_^2^ = 0.54; (target): *F*_(1,58)_ = 35.49, *p* < 0.001, η_p_^2^ = 0.38, but no interaction effect *F*_(2,116)_ = 0.53, *p* = 0.559, η_p_^2^ = 0.009. Planned contrasts revealed reduced capture when the singleton distractor was presented at the high-probability location compared to a low-probability location (fixed-feature task: $\Delta M$_(high−low)_ =  − 27.36 ms, *SEM* = 3.66 ms, *F*_(1,29)_ = 55.80, *p* ≤ 0.001, η_p_^2^ = 0.66; mixed-feature task: $\Delta M$_(high−low)_ =  − 29.25 ms, *SEM* = 6.96 ms, *F*_(1,29)_ = 17.64, *p* =  ≤ 0.001, η_p_^2^ = 0.38). However, responses were still slower with distractors presented at the high-probability location than responses in distractor-absent trials (fixed-feature task: $\Delta M$_(high−absent)_ = 11.27 ms, *SEM* = 3.59 ms, *F*_(1,29)_ = 9.83, *p* = 0.004, η_p_^2^ = 0.25; mixed-feature task: $\Delta M$_(high−absent)_ = 16.77 ms, *SEM* = 4.36 ms, *F*_(1,29)_ = 14.78, *p* = 0.001, η_p_^2^ = 0.34; see Fig. 2a). Inspection of error rates indicated no speed-accuracy trade-off.

### Learned suppression in distractor-absent trials

To assess whether the high-probability location was suppressed also in distractor-absent trials, that is, irrespective of distractor presence, RTs were also analysed in distractor-absent trials. To this end, mean RTs were submitted to a two-way mixed-design ANOVA with the within-participants factor target location (target presented at high-probability vs. low-probability distractor location) and the between-participants factor target (fixed-feature target, mixed-feature target). Responses were slower for targets presented at the high-probability location (*M* = 696.23 ms, *SEM* = 13.75 ms) compared to targets presented at the low-probability locations (*M* = 683.46 ms, *SEM* = 11.47 ms; *F*_(1,58)_ = 6.17, *p* = 0.016, η_p_^2^ = 0.096; see Fig. 2b), and faster in the fixed-feature (*M* = 634.01 ms, *SEM* = 9.34 ms) than in the mixed-feature task (*M* = 745.69 ms, *SEM* = 11.36 ms) *F*_(1,58)_ = 30.37, *p* < 0.001, η_p_^2^ = 0.34. The interaction was not significant *F*_(1, 58)_ = 0.13, *p* = 0.717, η_p_^2^ = 0.002.

### Intertrial location priming

To examine the possibility that the reported effects resulted at least in part from trial-by-trial priming rather than from distractor location learning, the data was re-analysed without trials in which the distractor location was repeated from the previous trial (*n* − 1)^12,24^. The RT difference observed between trials with distractors at high-probability and low-probability locations remained significant (fixed-feature task *t*(29) = 7.2, *p* =  < 0.01*;* mixed-feature task *t*(29) = 3.54, *p* =  < 0.01), ruling out that the effect could be entirely driven by intertrial priming.

### The shape of the spatial suppression gradient

**Figure 3:**
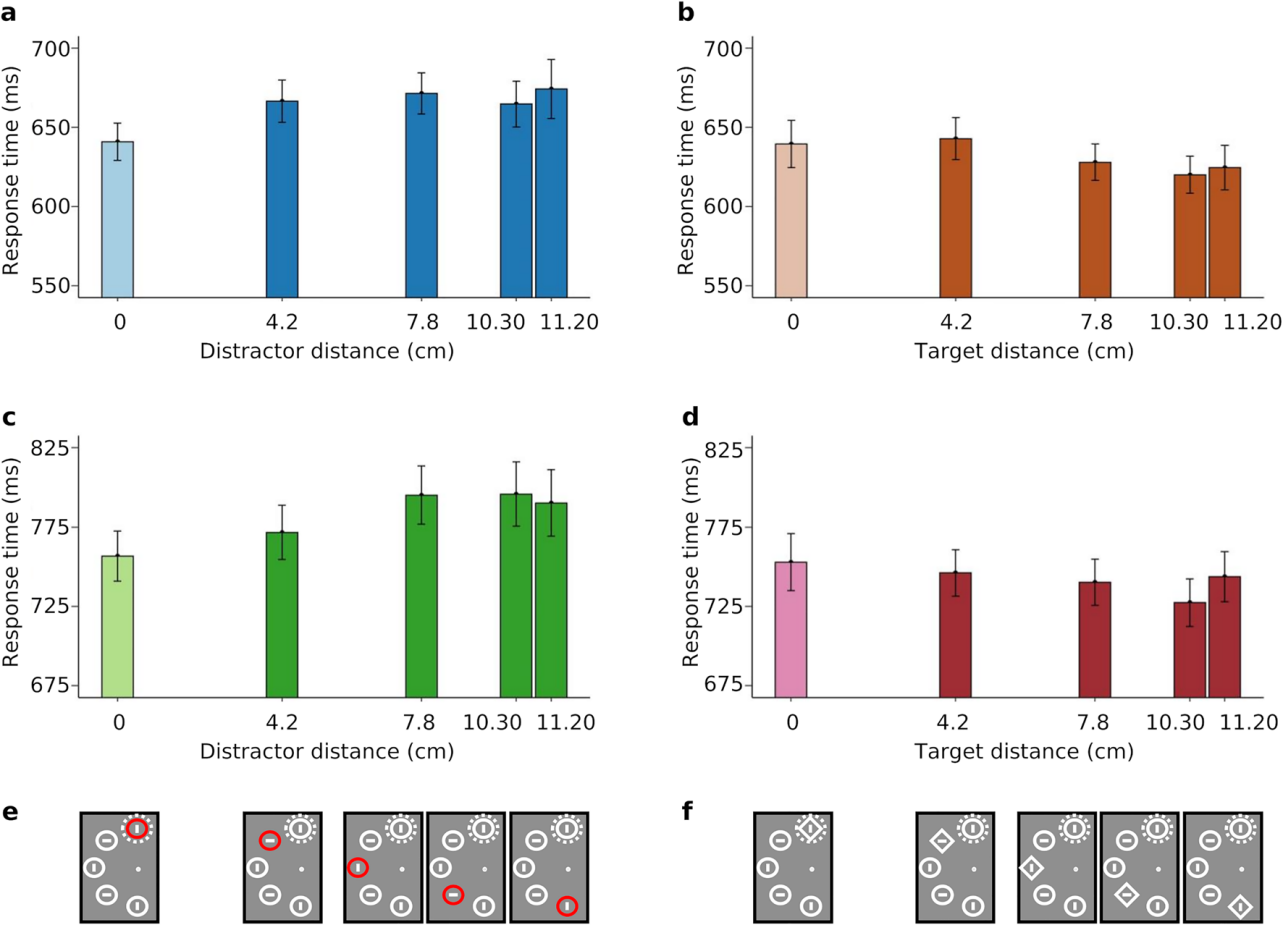
Response time results plotted by distance to the high-probability location, separately for the fixed-feature task (top row) and the mixed-feature task (bottom row). (**a**, **c**) Bar graphs show mean response times as a function of the distance of the distractor to the high-probability location (distance 0), with the distractor presented at the high-probability location (light blue and green) or the low-probability locations (dark blue and green). (**b**, **d**) Bar graphs show mean response times as a function of the distance of the target to the high-probability distractor location (distance 0) in distractor-absent trials, with the target presented at the high-probability distractor location (light orange and pink) or the low-probability distractor locations (dark orange and pink). Error bars show standard errors of the means. (**e**, **f**) Examples of the visual search displays illustrate the corresponding distractor location (**e**) and the location of the target (**f**) in a trial (the displays are only partially shown). The high-probability location is shown on the top vertical and indicated by the dotted line.

Figure 3 depicts the spatial gradient of suppression visualised by plotting RTs as a function of the Euclidean distance to the high-probability location of either the distractor (in distractor-present trials) or the target (in distractor-absent trials). The response times increase with increasing distractor distance from the high-probability location (Fig. 3, left panels), and decrease with increasing target distance (Fig. 3, right panels). For a more detailed quantification of these response time modulations, we estimated a hierarchical Bayesian model based on the idea that suppression decays exponentially with distance to the high-probability distractor location. The model includes three parameters: (1) spatial gradient of suppression: the rate at which suppression decreases across the visual field; (2) maximum (potential) capture at locations where suppression has faded out; (3) residual capture at the high-probability location. The latter two parameters were set to come from a common distribution for both tasks and were partially informed by measurements from similar experiments reported in the literature (see Methods and Supplementary Information A for details). The spatial gradient of suppression was not found clearly different between tasks (0.06, 95% HPD [− 0.02, 0.17], zero within the interval), indicating that the difference might be absent or subtle and the power in the present analysis not sufficient to detect it.

**Figure 4:**
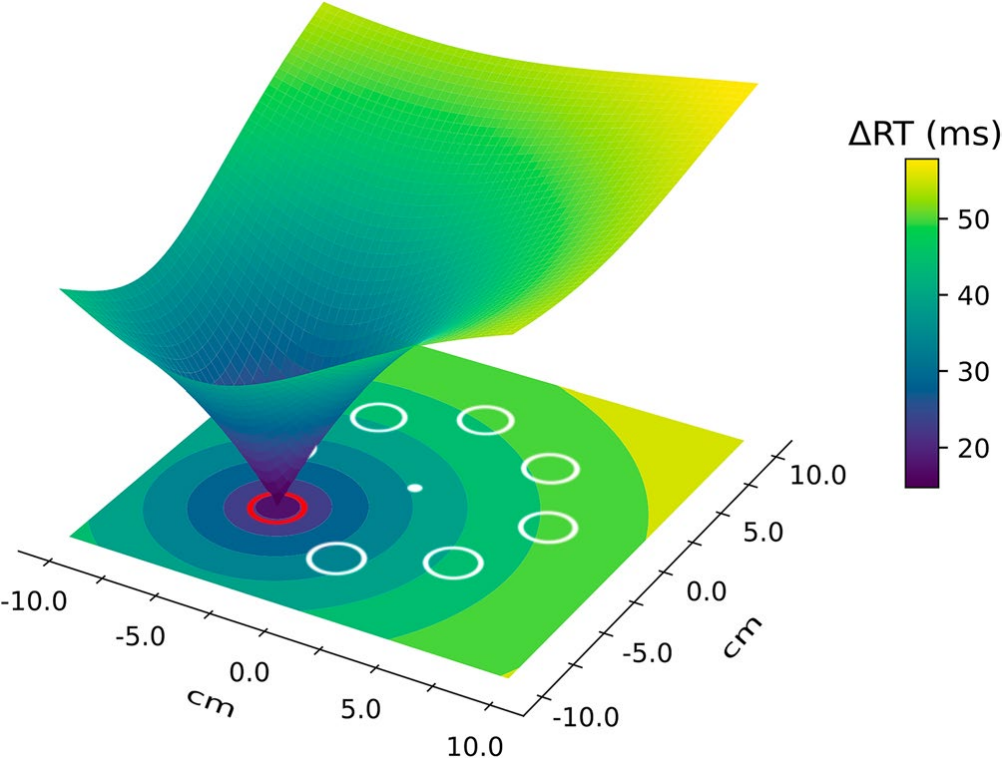
Visualization of the suppression map. The colours show the estimates of the spatial gradient of suppression. The high-probability location is indicated by the colour distractor.

To provide a visual impression of how the distractor suppression distributes spatially across the display, we computed a visualization of the suppression map by having the spatial suppression radiate outward from the high-probability location with the estimated parameters (see Fig. 4). Because the difference in the spatial gradient between the two tasks was rather uncertain (0.06 [− 0.02, 0.17]), the visualization was based on the mode of the averaged posteriors from both tasks (spatial suppression gradient: 0.11, maximum capture at locations where suppression has faded out: 64 ms, residual capture at the high-probability location: 13 ms); the highly similar specific visualizations and posterior modes for each condition can be seen in the Supplementary Information A, Fig. S4 and Table S1.

Note that this suppression map does not necessarily need to be understood as a map implemented by the visual system. First and foremost, it is a visualization of how much suppression a stimulus would receive if it was repeatedly presented at a certain location in the visual field. However, it can be understood as a selection-history-based map that encodes experience-based suppression within the visual system. In the Supplementary Information A, Fig. S5, we show how it could combine with other maps of the visual system to suppress distractor stimuli in experimental settings as ours.

### Learning curves

**Figure 5:**
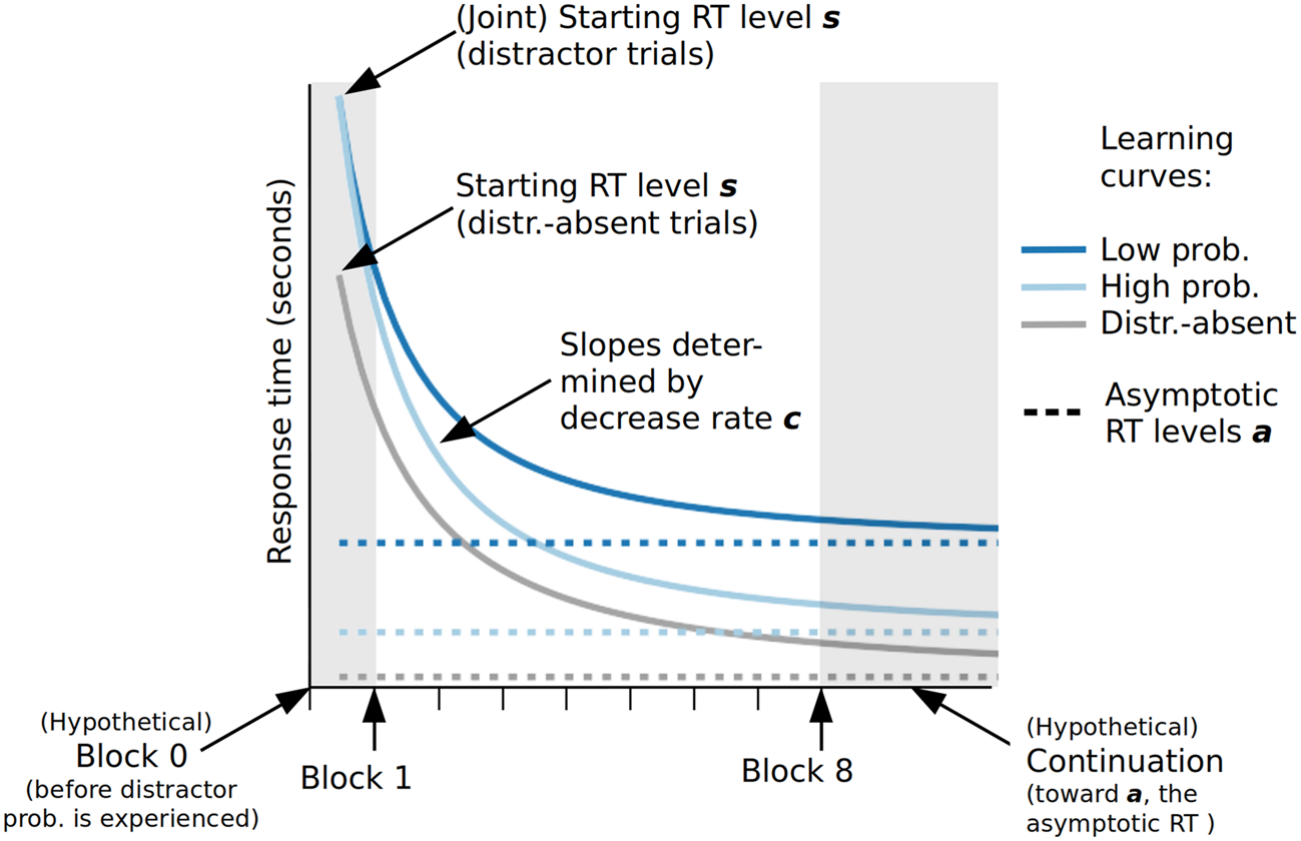
Illustration of the learning curve model. The annotations in the figure highlight the measurement range (Block 1 to 8) and visualize the model parameters.

A hierarchical Bayesian model was implemented to assess the time course of distractor location learning over the successive experimental blocks. The model allowed quantifying distractor location learning as different learning curves for the three distractor conditions (distractor-absent baseline, distractors at the high-, and at low-probability locations). The model includes the following parameters (see also Fig. 5): (1) the starting response time (RT) level *s*, with which participants initially perform when they start working on the task, (2) the asymptotic RT level *a,* toward which performance converges in the long run, and (3) a decrease rate *c* that models how quickly the asymptotic RT level is approached. For each task, the starting response time *s* was implemented as a common parameter for the high- and low-probability distractor conditions, but as a separate parameter for the distractor-absent baseline. This was done to take into account that initially the same RTs can be expected for the distractor-present trials, since the spatial bias needs to be learned before differences can evolve. Note that the starting RT level *s* is modelled at an imaginary 0th block, which represents the hypothetical response time before the experiment started. Consequently, despite their common origin, even the high- and low-probability learning curves can differ already after the first experimental block. By estimating the asymptotic response time levels *a* relative to the distractor-absent baseline, it becomes possible to measure the asymptotic distractor cost in the low- and high-probability locations after the observer has sampled and learned the different distractor frequencies over sufficient time.

By looking at the decrease rates *c* relative to the baseline (*decrease rate differences*), the model can reveal whether these asymptotes are reached at different speeds.

As a first check of whether distractor location learning influences the asymptotic RT level *a*, the decrease rate *c*, both, or neither, we run a Bayesian model comparison using Pareto-smoothed importance sampling leave-one-out cross-validation^40,41^ (see Methods and Supplementary Information B for details). The model without any effects of distractor location learning ranked worst for both tasks. The model that only includes the decrease rate effects only reached the third rank for both tasks. The “asymptotic RT level effect” and the “both-effects model” performed better, ranking first (“asymptotic RT level”) and second (“both-effects model”) in both tasks (see Supplement Table S2 for details). This suggests that the model needs to account for the differences in the asymptotic RT levels that are reached in distractor location learning. Given the small difference between the top two model versions (see Supplement Table S2) it remains somewhat unclear whether the decrease rate differences contribute to explaining the data. Hence, for the subsequent parameter estimation we conservatively employed the most general model (i.e., the “both-effects model”) that allows for differences in both, the asymptotic RT levels and the decrease rates.

**Figure 6:**
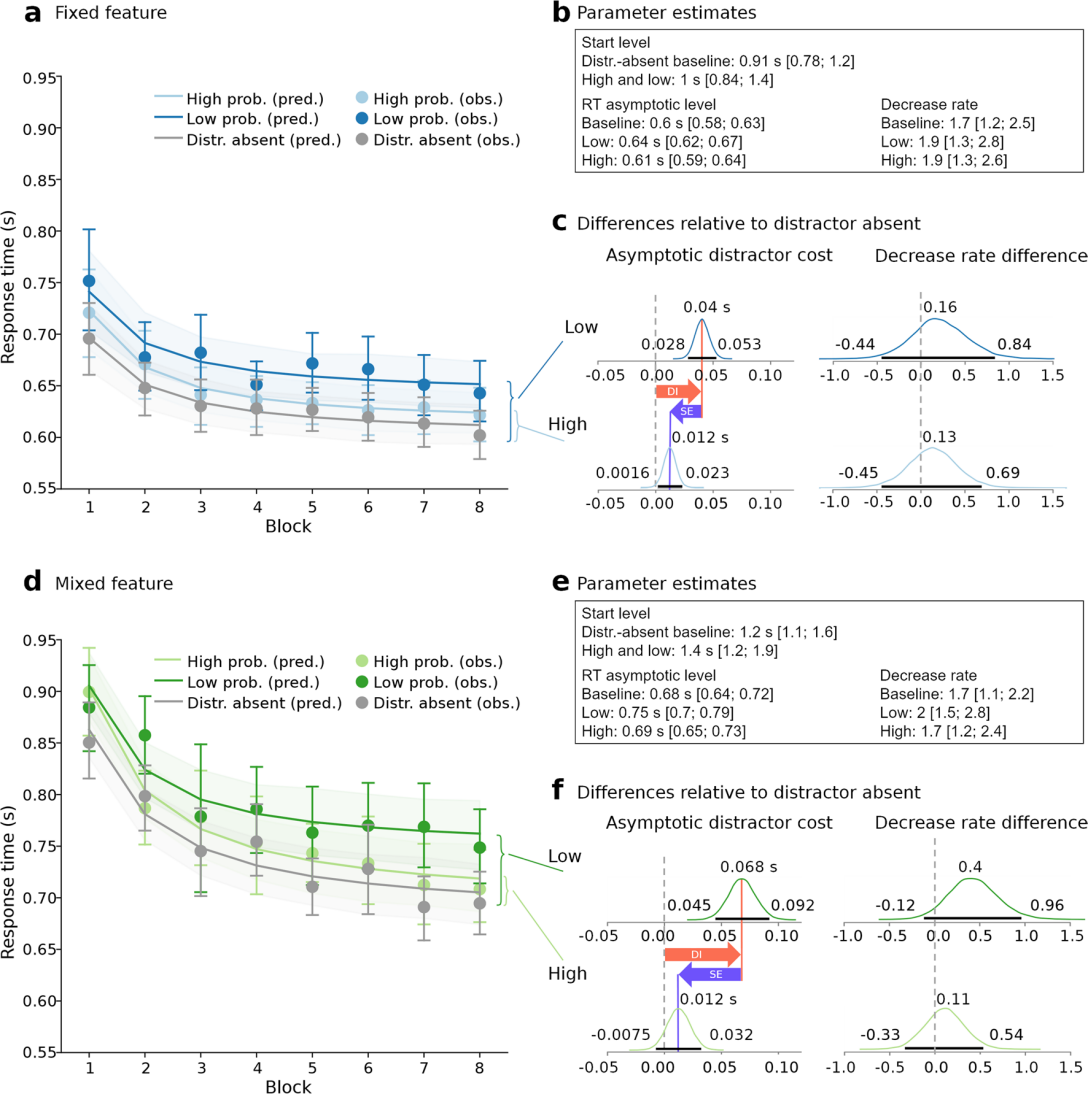
Learning curves across experimental blocks for the fixed-feature task (**a–c**) and mixed-feature task (**d–f**). (**a**, **d**) Points show the observed response times for the distractor-absent condition (in grey), and low- (dark colours) and high-probability (light colours) locations. Error bars are 95% confidence intervals. Lines visualize the model predictions as the mean of the expected RT of each participant at each level (means of the posterior predictive distributions). The shaded areas depict the 95% confidence intervals of these participant scores. Block 1 is the practice block. (**b**, **e**) Group-level parameter estimates with 95% Highest Probability Density (HPD) range in square brackets. (**c**, **f**) Posterior distributions of the asymptotic distractor cost (left panels; low- and high-probability locations in upper and lower rows, respectively) and decrease rate differences to the baseline (right panels). The orange arrows depict the asymptotic distractor interference (DI), the purple arrows depict the asymptotic distractor suppression effects (SE). Horizontal black bars indicate the 95% HPD range.

Figure 6 depicts the estimated learning curves (Fig. 6a, d), the group-level parameter estimates (Fig. 6b, e), and relative parameter differences to the distractor-absent baseline (Fig. 6c, f).

We first report the modes of the group-level posterior distributions of the between-task differences (exemplarily for the distractor-absent baseline) and their 95% Highest Posterior Density (HPD) intervals in square brackets. These differences are not shown explicitly in Fig. 6. As mentioned earlier, the overall response times were faster in the fixed-feature task compared to the mixed-feature task. Visually, these differences between tasks manifest in the different vertical levels in which the learning curves reside when comparing Fig. 6a, d. The curves from the fixed-feature task reside at substantially lower levels than the ones from the mixed-feature task. This is reflected in the different starting RT levels *s* ($\Delta s_{{{\text{between}}}}^{{\text{absent}}} = s_{{{\text{mixed}}}}^{{\text{absent}}} - s_{{{\text{fixed}}}}^{{\text{absent}}} = 320\;{\text{ms}}\;\left[ {110, 590} \right]$) and asymptotic RT levels *a* ($\Delta a_{{{\text{between}}}}^{{\text{absent}}} = a_{{{\text{mixed}}}}^{{\text{absent}}} - a_{{{\text{fixed}}}}^{{\text{absent}}} = 76\;{\text{ms}}\;\left[ {47, 98} \right]$) between tasks. Both lie far from zero in the positive range, indicating substantially smaller starting and asymptotic RTs in the fixed-feature task.

Second, we turn to the differences in the asymptotic RT levels and the decrease rates relative to the baseline within each task (calculated at the participant level).

In the fixed-feature task, the asymptotic distractor cost in the low-probability condition was estimated at $\Delta a_{{{\text{fixed}}}}^{{\text{low}}} = a_{{{\text{fixed}}}}^{{\text{low}}} - a_{{{\text{fixed}}}}^{{\text{absent}}} = 40\;{\text{ms}}\;\left[ {28, 53} \right]$ (cf. Fig. 6c, upper left panel). In the high-probability condition, the asymptotic distractor cost was substantially smaller with $\Delta a_{{{\text{fixed}}}}^{{\text{high}}} = a_{{{\text{fixed}}}}^{{\text{high}}} - a_{{{\text{fixed}}}}^{{\text{absent}}} = 12\;{\text{ms}}\;\left[ {1.6, 23} \right]$ (cf. Fig. 6c, lower left panel). Turning to the decrease rates *c*, the difference in decrease rates between the distractor-absent baseline and both the low- and high-probability conditions was estimated at similar magnitudes with $\Delta c_{{{\text{fixed}}}}^{{\text{low}}} = c_{{{\text{fixed}}}}^{{\text{low}}} - c_{{{\text{fixed}}}}^{{\text{absent}}} = 0.16 \left[ { - 0.44, 0.84} \right]$ and $\Delta c_{{{\text{fixed}}}}^{{\text{high}}} = c_{{{\text{fixed}}}}^{{\text{high}}} - c_{{{\text{fixed}}}}^{{\text{absent}}} = 0.13{ }\left[ { - 0.45, 0.69} \right]$; indicating faster approach to their asymptotes than in the baseline (cf. Fig. 6c, right panels).

In the mixed-feature task, the asymptotic distractor cost was large with $\Delta a_{{{\text{mixed}}}}^{{\text{low}}} = a_{{{\text{mixed}}}}^{{\text{low}}} - a_{{{\text{mixed}}}}^{{\text{absent}}} = 68\;{\text{ms}}\;\left[ {45, 92} \right]$ in the low-probability condition whereas it was much smaller in the high-probability condition ($\Delta a_{{\text{mixed}}}^{{\text{high}}}$ difference estimated at $12\; {\text{ms }}\left[ { - 7.5, 32} \right]$) (cf. Fig. 6f, left panels).

The difference in decrease rates was estimated at $\Delta c_{{\text{mixed}}}^{{\text{low}}} = c_{{\text{mixed}}}^{{\text{low}}} - c_{{\text{mixed}}}^{{\text{absent}}} = 0.4{ }\left[ { - 0.12, 0.96} \right]$ in the low-probability condition (cf. Fig. 6f, upper right panel), reflecting a higher decrease rate difference as in the fixed-feature task. In the high-probability condition, $\Delta c_{{{\text{mixed}}}}^{{\text{high}}}$ was estimated at $c_{{{\text{mixed}}}}^{{\text{high}}} - c_{{{\text{mixed}}}}^{{\text{absent}}} = 0.11{ }\left[ { - 0.33, 0.54} \right]$ (cf. Fig. 6f, lower right panel), reflecting a similar decrease rate difference as in the fixed-feature task.

### Reports of distractor location regularity

30% of the participants in the fixed-feature task and 20% in the mixed-feature task reported noticing regularities in distractor location. The probability with which these participants indicated the correct high-probability location was about chance level (11.11% fixed-feature task, 16.6% mixed-feature task).

## Discussion

We investigated distractor location learning in two variants of the additional singleton search task that differed in their predefined targets. Participants were asked to search for either a fixed-feature target (diamond) or a mixed-feature target (diamond or circle). When searching for a fixed-feature target, they can use a feature-specific target template, while when searching for a mixed-feature target, they can only use a coarse template that, at best, specifies that the target is a singleton in the shape dimension. Previous studies reported that a salient distractor is more likely to cause interference when observers search for a mixed-feature target with the target features changing unpredictably across trials when compared to search for a fixed-feature target where the features remain predictable (e.g., Burra and Kerzel^42^; Lamy and Yashar^43^; Leber and Egeth^44^; Pinto et al.^45^). We thus expected that observers would be less prone to capture with a feature target, and speculated that distractor location learning might be less beneficial and therefore reduced in the fixed-feature compared to the mixed-feature task.

Our results replicated previous findings that showed reduced attentional capture when the distractor was presented at a highly probable location compared to the other, less probable locations^12,13,24–26^. Suppression effects were observed as reduced distractor interference when the distractor was presented at the high-probability distractor location, but also as a response time increase when the target was presented at this location. The effect on the target suggests that learned spatial suppression is feature-blind (but see Allenmark et al.^28^; van Moorselaar et al.^12^; Stilwell et al.^31^). According to the post-experimental survey, the spatial regularity went unnoticed by most of the participants.

These results seem to suggest that distractor location learning was not modulated by the precision of the target template, which would be consistent with previous findings^29,32^. However, these studies by Ivanov and Theeuwes^29^ and by Wang and Theeuwes^32^ did not only manipulate the targets types, but also other parameters of the visual search displays which might have affected the salience signals^35,46^. In the present study, we eliminated this issue, by keeping the distractor, non-targets, and the number of items in the search displays constant between tasks. One reason for the absence of the expected difference between tasks might be that differences were too subtle and not visible on the level of averages across the whole course of the experiment.

To follow up on this, we conducted a learning-curve-based analysis of the time course of distractor location learning. The learning curves allowed assessing the asymptotic distractor costs for the high- and low-probability condition in both tasks, and how quickly they were approached.

Recall that distractors in the low-probability condition appear unpredictably at one of 7 locations, hence participants cannot benefit from distractor location learning in these trials. The asymptotic distractor cost in this condition is thus an estimate of the distractor interference *DI* (see orange arrows in Fig. 6 for visualization). Comparing *DI*s across tasks reveals that distractor interference was 40 ms [28, 53] in the fixed-feature task and thus substantially lower than in the mixed-feature task with 68 ms [45, 92]). Subtracting the distributions yields a difference of 25 ms [− 0.18, 54]). This finding fits well with the assumption that a fixed-feature target is less prone to distractor interference than a mixed-feature target. It also supports the notion that a more precise target template allows better down-weighting of the irrelevant distractor dimension^10,12^.

To analyse whether distractor location learning was still effective in the fixed-feature task, we estimated the suppression effect that emerged with distractor location learning (purple arrows in Fig. 6). We found substantial distractor suppression in both tasks (mixed-feature task: 55 ms [36, 75]; fixed-feature task: 28 ms [18, 38]), yet the distractor suppression effect was smaller in the fixed-feature task (29 ms [4.1, 51]). Thus, a fixed-feature target seems to not only enable down-weighting of the distractor dimension when feature overlap is prevented, but also to reduce the need for distractor location learning.

Our spatial inhibition model allowed us to quantify the spatial distribution of distractor suppression by assuming that suppression radiates outward from the distractor location, following an exponential decline over distance. As illustrated in Fig. 4, stimulus locations close to the high-probability distractor location are also influenced notably by distractor suppression, and even those further away receive some inhibition. This finding is interesting, as one might expect suppression to be more precisely localized. For instance, Chelazzi et al.^47^ found priority map modulations induced by reward probabilities to be precisely localized at the stimulus locations that were associated with higher or lower frequencies of high-reward targets. Note that the broad funnel of suppression over large parts of the display in our map is not an artefact of our modelling approach: A decay of suppression with increasing distance to the distractor is also visible in the averaged RT data plotted in Fig. 3. Possibly, the occasional presence of distractors at the low-probability locations prevented the formation of a steeper, more precisely tuned suppression gradient. The issue could be addressed in future studies.

Although we interpret our findings as an index of spatial suppression, also other interpretations might be effective in designs with a frequency bias. As the distractor was presented less often at the low-probability than at the high-probability location, also rarity^48^, novelty^49,50^ or local distractor frequency^51–55^ might have contributed to the present findings. The asymptotic RT level at the low-probability locations might thus be higher than in a design with a balanced distractor location probability.

Another interesting aspect is that some asymptotic distractor costs remain for distractors at the high-probability location, where the RT deficit is about 12 ms in both tasks. We consider this a consequence of our task design: as the target was presented occasionally at that location, the high-probability distractor location could not be completely ignored. Thus, even in the long-run, spatial suppression might not reach the distractor-absent baseline level. Interestingly, also the “experience-based tuning” account by Vecera et al.^56^ does not assume that attentional control becomes purely goal-driven with learning, and that salient distractors no longer capture the observer’s attention. The authors argue that simple tasks with homogeneous non-targets and a singleton target can increase the distractor’s overall salience to an extent that will render it impossible to ignore it completely.

The pattern of decrease rates in the different distractor conditions and tasks (relative to the distractor-absent baseline) was rather indifferent, with three out of four rates estimated at roughly similar levels (0.11 to 0.16), indicating that the distractor learning curves converged slightly faster compared to the distractor-absent baseline. One estimate, namely that of the low-probability location in the mixed-feature task, deviated somewhat from this pattern: the decline was substantially faster than in the distractor-absent baseline (a rate of 0.4). Low-probability location trials in the mixed-feature task also showed the largest asymptotic distractor cost. A tentative explanation for the decrease rate being higher in this condition could be that little can be learned when the target features and the spatial location of the distractor vary unpredictably across trials. The small possible improvement is reached quickly, whereas with a fixed-feature target further improvements can accumulate over time. Hence, the final performance level is reached more quickly than in other conditions, but it remains on a higher asymptotic response time level.

As noted by an anonymous reviewer, the differences in the learning curves would presumably have turned out larger with a stronger manipulation of the target template, for instance, with differently shaped non-target items. However, manipulating the non-target heterogeneity might have not only changed distractor salience and distractor interference^34,57^, but also the extent of distractor location learning. To eliminate these effects and to focus on the precision of the target template on distractor location learning, which was the core of this study, we varied only the target between tasks while the distractor and non-target features were kept constant. A comparison between search arrays with homogeneous and heterogeneous non-targets remains a topic for future research.

In sum, the estimates of the asymptotes of the learning curves show a pattern very compatible with our hypotheses: distractor interference is smaller in the fixed-feature task, in which participants can use a more precise target template. Also, less distractor location learning (reflected in a smaller asymptotic suppression effect) is at work in the fixed-feature task to reach the same asymptotic performance level as in the mixed-feature task. Detecting the effects in the asymptotes means that they evolve late over the course of the experiments. This could well be the reason that we did not detect these patterns in the RTs when averaged over the entire experiment.

Distractor location learning which evolves over time seems to fit well into the “experience-based tuning” account^56^. Vecera et al.^56^ propose that stimulus-driven and goal-driven control lie on a continuum, with their relative contributions shifting over time through learning. That is, the observers’ attention is more stimulus-driven at the beginning of the task and goal-driven control develops over time with task learning. From this perspective, differences between the fixed-feature and the mixed-feature task should evolve with increasing task experience, namely when observers learn to apply a precise, feature-specific target template in the feature task, allowing them to perform goal-directed control more efficiently. This is exactly what we see in our data. Moreover, also the efficiency of target selection benefits from the observer’s exposure to a particular distractor and its features^56^ (see also Vatterott and Vecera^58^). The more participants learn about the distractor location frequencies in performing the task, the better they manage to suppress the distractor at the high-probability location.

From a broader perspective, our results can also be interpreted in terms of predictive processing^59,60^. From this perspective, salient distractors inevitably capture attention because they emit a strong signal that the brain predicts to be highly precise by default. In the fixed-feature task, the target features are highly predictable. Thus, participants can upregulate the precision of the target signal, thereby reducing the precision signal associated with the distractor. The increased precision of the target signal reduces the sensitivity for attentional capture and thus the need for distractor location learning. In the mixed-feature task, by contrast, target and distractor features overlap, which prevents weighting of the precision signal. Consequently, distractor location learning is helpful to reduce the salience signal of the distractor.

To conclude, the present study adds to a growing body of literature demonstrating that attentional capture by salient but task-irrelevant information can be reduced based on learning of spatial distractor regularities. The modulation of distractor location learning by the precision of the target template does not necessarily manifest on the level of average performance (RTs averaged over the whole course of the experiment). However, a fine-grained perspective based on the time course of distractor location learning suggests that there are differences that depend on the target template. Highly predictable target features that allow for a more precise target template require less suppression of the distractor. As a consequence, distractor location learning is less prominent in the fixed-feature task, possibly because it is less needed to find the target efficiently.

## Methods

### Participants

Participants were recruited via Prolific (https://www.prolific.co) or via the local research participation system SONA Systems, and participated for monetary compensation (£7.66/hour) or course credit. Nationality was not pre-screened leading to heterogenous samples. Informed consent was obtained from each participant prior to the experiment. Fifteen out of 75 participants had to be excluded (3 of them in the fixed-feature task) due to low accuracy (< 50% in more than one block), slow response times (> 2.5 SD) or no responses in the practice block. The remaining sample consisted of a total of 60 participants, 30 participants (18 female, 12 male; mean age 23.5 years, range 18–30 years) in the fixed-feature task, and 30 participants (16 female, 14 male; mean age 21.9 years, range 18–28 years) in the mixed-feature task. All participants were naïve to the design and objective of the experiment and reported normal or correct-to-normal visual acuity and colour vision. The experiment was approved by the local Ethics Committee of the Department of Psychology at Philipps-University Marburg and conducted in accordance with the ethical principles of the Declaration of Helsinki.

### Apparatus and stimuli

The experiment was set up for online data collection by using the JavaScript library jsPsych^61^. Stimulus sizes were controlled using the jsPsych-resize plugin. The plugin displayed a square that participants were asked to resize until it was the size of a credit card (or a card of the same size) held up to the screen. In this way, a scaling factor for the subsequent stimuli was set, which ensured that the stimuli were displayed at the specified size regardless of individual screen sizes and resolutions^62^.

The stimuli and tasks are illustrated in Fig. 1a. All stimuli were presented on a dark grey background (RGB 143, 143, 143) and were arranged circularly (radius: 5.6 cm) around a central fixation point (RGB 255, 255, 255, radius: 0.15 cm). The circle-shaped stimuli were 1.25 cm in radius and the diamond-shaped stimuli were 2.3 × 2.3 cm. Each stimulus contained a grey line (RGB 255, 255, 255, 1.3 × 0.1 cm). Stimulus outlines were also grey (RGB 255, 255, 255), except for the outline of the colour singleton distractor which was randomly assigned from two out of three colours (red, green, blue). To ensure that participants could discriminate the singleton distractor from the grey stimuli on their own desktop devices, a colour-array was shown prior to the experiment. The colour-array displayed the selected two distractor colours in four different hues (see Fig. 1b, red: RGB 255, 0, 64; RGB 255, 0, 0; RGB 255, 64, 0; RGB 255, 128, 0; green: RGB 128, 255, 0; RGB 64, 255, 0; RGB: 0, 255, 0; RGB 0, 255, 64; blue: RGB 0, 191, 255; RGB 0, 128, 255; RGB 0, 64, 255; RGB 0, 0 255). Participants were asked to choose a hue for each colour which was well visible among the grey stimuli on their screen. The chosen hues became the colours of the singleton distractor in the experiment.

### Procedure and design

In both search task variants, a trial started with a fixation point which remained on screen throughout a trial. After 600 ms, the visual search display appeared for 200 ms, followed by the response display with the fixation point. Participant’s task was to report the orientation of the line (horizontal vs. vertical) in the shape target as quickly and as accurately as possible. If the response was incorrect or too slow (> 1200 ms), a warning message was shown for 1000 ms. After an intertrial interval of 500–700 ms, the next trial started. The experiment consisted of one practice and seven experimental blocks á 48 trials. After every 24 trials, the participants could take a break. To allow participants to refocus after a break, a five seconds countdown was set up before the task continued. Mean response times and mean accuracy were displayed after each block.

In 50% of the trials, a colour distractor was shown. In two-thirds of the distractor-present trials, the distractor was presented at one of the eight possible display locations (high-probability location), and in the remaining one-third of trials at one of the other locations with equal probability (low-probability locations). The high-probability location was randomly assigned and remained unchanged during the experiment. The target was presented with equal probability at each location in distractor-absent and low-probability trials. In high-probability-trials, the target was presented randomly at the locations not occupied by the distractor. At the end of the experiment, participants were asked whether they had noticed that the colour distractor occurred more often at one location. Participants who reported having noted the regularity, were asked to indicate the high-probability location in the search display.

### Response time analysis and error rates

Trials in which participants responded incorrectly, failed to respond or responded in less than 200 ms (11.82% fixed-feature task; 26.27% mixed-feature task) were removed from analyses. Trials with response times above or below 2.5 SD from each participant’s mean response time calculated separately for each condition per experimental block were excluded (1.54% fixed-feature task; 0.97% singleton condition). Blocks with an accuracy below 50% were removed from analyses (one block fixed-feature task; 3 blocks mixed-feature task). Mixed two-way ANOVAs were calculated followed by simple contrasts using IBM SPSS Statistics 27. Assumption of normality was tested (Shapiro–Wilk) and fulfilled. If sphericity was violated, Greenhouse–Geisser corrected *p*-values and uncorrected degrees of freedom are reported. For the Bayesian model comparison, exclusion criteria were stricter to determine distractor location learning across blocks. Here, participants whose accuracy dropped below 50% in only one block (including the practice block) were excluded (one participant fixed-feature task; five participants mixed-feature task).

### Spatial inhibition

To quantify the spatial gradient of distractor suppression, mean RTs were analysed as a function of the Euclidean distance of the distractor to the high-probability location (see also Wang and Theeuwes^63^). Distance 0 cm corresponds to the distractor being presented at the high-probability location, the distances 4.25 cm, 7.85 cm, 10.30 cm and 11.20 cm to the locations 45°, 90°, 135° and 180° away from the high-probability location, respectively. An exponential growth function computing ΔRTs (distractor-present trials—distractor-absent trials) as a suppression curve (SC) was embedded in a hierarchical Bayesian model (see Supplementary Information A for details):

1 $$ \Delta {\text{RT}}_{{\text{i}}} \left( x \right) \sim {\text{Normal}}\left( {\mu = \left( {1 - {\text{e}}^{{\left( {{-}r_{i} x} \right)}} } \right)  \left( {m_{i} - d_{i} } \right) + d_{i} , \, \sigma = sd_{i} } \right) $$

where ΔRT for each participant *i* at distance *x* is calculated by the spatial suppression gradient *r*, the maximum capture at locations where suppression has faded out *m* and the residual capture at the high-probability location *d*.

Posterior predictive samples were computed as described in the previous section. The estimated distribution modes were used to build a novel data-driven 3D representation of suppression.

### Learning curve model

To investigate distractor location learning across experimental blocks, a hierarchical Bayesian model was applied which modelled the response times as a function of the distractor location with a learning curve (LC; see Supplementary Information B for details; cf. Bergmann et al.^39^):

2 $$ {\text{RT}}_{i}^{j} \left( x \right) \sim {\text{Normal(}}\mu { } = a_{i}^{j} + \left( {s_{i}^{j} - a_{i}^{j} } \right)\left( {x + 1} \right)^{{{-}c_{i}^{j} }} , \, \sigma = sd_{i} ) $$

where the response times (RT) for the distractor location conditions *j* and for each participant *i* in block *x* are calculated by the asymptotic RT level *a* at which the slope of the curve approaches zero, the starting RT level *s* and the decrease rate parameter *c*. The distractor-absent trials were determined to be the reference. The starting response time *s* was implemented as a common parameter for the high- and low-probability distractor conditions, but as a separate parameter for the distractor-absent baseline and was set before the start of the practice block. Here, the practice block was included in the analysis while it was excluded for all other reported analyses.

The model comparison considered four different assumptions:

1. Both effects: Distractor location learning affects both the decrease rate parameter and the asymptotic RT levels
2. Decrease rate effect: Distractor location learning affects the decrease rate parameter, but not the asymptotic RT levels
3. Asymptotic RT level effect: Distractor location learning affects the asymptotic RT levels, but not the decrease rate parameter
4. No effect: Distractor location learning neither affects the decrease rate parameter nor the asymptotic RT levels

Parameter estimates are based on posterior predictive samples drawn using the Python library PyMC v4^64^ to draw 20,000 Markov chain Monte Carlo samples (in 4 chains) with No-U-Turn Sampler^65^. These samples were subsequently thinned, keeping only every 4th sample to speed up computations and reduce required storage space. The model comparison was performed by using the Python library ArviZ^41^ which compared the models based on Pareto-smoothed importance sampling leave-one-out cross-validation^40^. The code that implements the model can be found in the repository linked below. Note that an earlier version of the model with a hierarchical structure analogous to the one in Bergmann et al.^39^, which did not employ correlated varying effects, was not able to provide sufficiently certain estimates of the differences between distractor conditions.

## Supplementary Information

Supplementary Information.

## Data Availability

The datasets analysed during the current study and the hierarchical Bayesian models are available at OSF: https://osf.io/5wcex/ and GitHub: https://github.com/AylinH/HanneEtAl2022_DisLocLearning.
