# Supplementary material for: Target templates and the time course of distractor location learning
Source: Sci Rep. 2023 Jan 30;13:1672. doi: 10.1038/s41598-022-25816-9 (PMC9886952; doi:10.1038/s41598-022-25816-9)
Supplement: Supplementary file 1 — Supplementary Information. [file 41598_2022_25816_MOESM1_ESM.pdf]

# Supplementary Information

## Target templates and the time course of distractor location learning

**Aylin A. Hanne*, Jan Tünnermann, and Anna Schubö**

Cognitive Neuroscience of Perception and Action,
Department of Psychology, Philipps-University Marburg, Germany
*aylin.hanne@uni-marburg.de

## Supplementary Methods A: Spatial suppression gradient model

### A1: Model

To quantify how response times were modulated as a function of distractor distance $x$ to the high-probability location, and to assess potential differences between the fixed-feature and the mixed-feature task, we implemented an exponential spatial decay model.:

$$\text{suppression}(x, r_i, m_i, d_i) = \left(1 - \mathrm{e}^{(-r_i \cdot x)}\right) \cdot (m_i - d_i) + d_i$$

The three parameters are: (1) *r*: spatial gradient of suppression; models how fast suppression decreases across the visual field; (2) *m*: maximum (potential) capture at locations where suppression has faded out, and (3) *d*: residual capture at the high-probability location. The main parameter of interest was the spatial gradient of suppression because it could reveal whether there is a difference between tasks in how sharply a location is suppressed. As it is difficult to estimate the shape of the suppression curve from only four measurement points, we enhanced the model by including prior knowledge: An informative prior based on several similar visual search experiments reported in the literature was used for the maximum capture at locations where suppression has faded out (for details, see section *Literature-based prior on m* below). Moreover, except for the spatial gradient of suppression, a common group-level distribution across both tasks was used. That is, while the estimates of individuals in both tasks can vary to a certain degree, they were assumed to originate from the same distribution, which allows the model to partially pool information across participants ("shrinkage", see Kruschke & Liddell[1]). Only for the spatial gradient of suppression separate group-level distributions were implemented for the fixed-feature task and mixed-feature task. Taken together, this approach facilitates fitting the model on rather uncertain data and promotes that the variability between the tasks leads to a difference in the estimates of the spatial gradient of suppression. This difference was estimated at 0.06, slightly shifted away from zero towards a narrower suppression in the mixed-feature task. This however is rather uncertain, because zero, "no difference" was still within the 95% HPD [-0.02, 0.17]. The other parameters were estimated at 13 ms (parameter *d*) and 64 ms (parameter *m*). Notably, the value of the latter is relatively high compared to the estimates from the literature that informed this parameter's prior (29.6 ms).

*Note:* The code for the model implementation can be found at https://github.com/AylinH/HanneEtAl2022_DisLocLearning. Moreover, the traces sampled from the model are available at https://osf.io/5wcex/ and can be assessed using ArviZ[2] if more details are needed than presented here.

## A2: Model structure, priors, sampling parameters

Hyperpriors:

$$m_\mu \sim \text{Normal}(\mu = 29.6, \sigma = 19.6) \qquad m_\sigma \sim \text{Uniform}(0, 20)$$
$$d_\mu \sim \text{Normal}(\mu = 10, \sigma = 5) \qquad d_\sigma \sim \text{Uniform}(0, 5)$$
$$r_\mu{}^{j\in\{\text{fixed, mixed}\}} \sim \text{Half-Normal}(\sigma = 0.1) \qquad r_\sigma{}^{j\in\{\text{fixed, mixed}\}} \sim \text{Uniform}(0, 0.1)$$
$$\text{sd}_\mu \sim \text{Uniform}(0, 100) \qquad \text{sd}_\sigma \sim \text{HalfCauchy}(\beta = 0.1)$$

Parameters for each participant $i$:

$$m_{z_i}{}^{j\in\{\text{fixed, mixed}\}} \sim \text{Normal}(\mu = 0, \sigma = 1)$$
$$m^{j\in\{\text{fixed, mixed}\}} \leftarrow m_\mu + m_{z_i}{}^{j\in\{\text{fixed, mixed}\}} \cdot m_\sigma$$

$$d_{z_i}{}^{j\in\{\text{fixed, mixed}\}} \sim \text{Normal}(\mu = 0, \sigma = 1)$$
$$d^{j\in\{\text{fixed, mixed}\}} \leftarrow d_\mu + d_{z_i}{}^{j\in\{\text{fixed, mixed}\}} \cdot d_\sigma$$

$$r_{z_i}{}^{j\in\{\text{fixed, mixed}\}} \sim \text{Normal}(\mu = 0, \sigma = 1)$$
$$r^{j\in\{\text{fixed, mixed}\}} \leftarrow r_\mu + r_{z_i}{}^{j\in\{\text{fixed, mixed}\}} \cdot r_\sigma$$

$$\text{sd}_{z_i}{}^{j\in\{\text{fixed, mixed}\}} \sim \text{Normal}(\mu = 0, \sigma = 1)$$
$$\text{sd}^{j\in\{\text{fixed, mixed}\}} \leftarrow \text{sd}_\mu + \text{sd}_{z_i}{}^{j\in\{\text{fixed, mixed}\}} \cdot \text{sd}_\sigma$$

Observations:

$$\text{RT}_i(x) = \text{Normal}(\mu = \text{suppression}(x, r_i, m_i, d_i), \sigma = sd_i)$$

MCMC Sampling parameters (PyMC[3])

| | |
|---|---|
| Sampler: | NUTS[4] |
| Number of posterior predictive samples: | 10,000 |
| Number of iterations to tune: | 10,000 |
| Target acceptance rate: | 0.999 |
| Initialization method used: | adapt_diag |

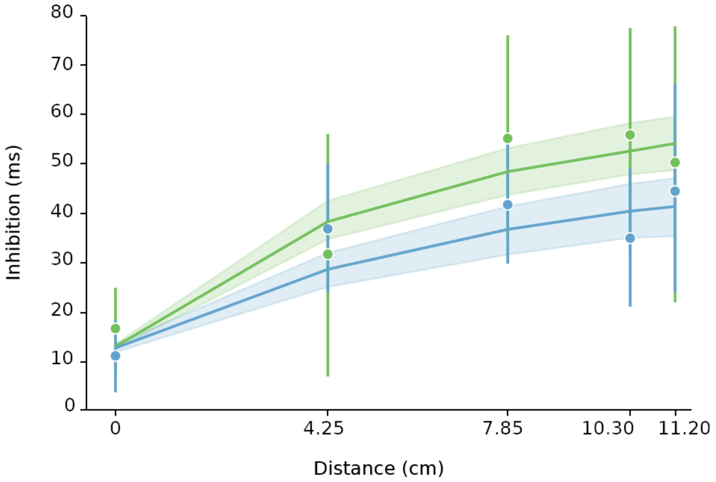


**Supplementary Figure S1.** Data and fits. Observed data (points) and predicted data as the mean of each participant's expected RT value at each level (means of the posterior predictive distributions of the RTs; solid lines) of the fixed-feature task and the mixed-feature task are shown in blue and green, respectively. The shaded areas depict the 95% confidence intervals over these participant scores. Response times are calculated relative to distractor-absent trials.

## A3: Literature-based prior on *m*

For most hyperparameters we could chose vaguely informative priors that let the data govern the outcome of the estimates. However, for the parameter *m*, which models the maximum performance decrease caused by a distractor farthest from the location that can be effectively inhibited, the prior needed to be more informative. The reason for this is that the location with the maximum performance decrease is not necessarily contained in the display. The largest distance between the high-probability location and a distractor in our displays was 11.20 cm and, hypothetically, it could still receive some inhibition radiating from the high-probability location. If there was a distractor somewhat farther away from the high-probability location, it might receive less inhibition and therefore have a higher impact on performance. As can be seen in Figure S1 it is not obvious whether the data points converge to a constant level of impairment or if the curves would continue to increase. This ambiguity is also present for the model and hence informative hyperpriors are required to regularize the fitting. To obtain information about the maximum performance decrease that might be observed with an uninhibited distractor, we sampled the literature on attentional capture to find research articles that met our criteria (target is a shape singleton, distractor is a colour singleton, 6–10 stimuli on the visual search display, no spatial learning, 30–70% distractor-absent trials). This approach allowed us to get an estimate of the observed maximum capture. We calculated the mean of these values and their standard deviation of reported attentional capture values (distractor present – distractor absent) in 14 research articles[5–18] (see Figure S2), which included a total of 36 experiments. We used these values as a hyperprior for the parameter $m_\mu$. Note that many of the values that informed these priors had to be read off from bar charts in the cited papers and hence might lack precision. Nevertheless, the purpose of the described procedure was to obtain a rough ballpark estimate of plausible values that can be fed into the prior. For the hyperprior $m_\sigma$ (which models the dispersion in the population), we chose a uniform distribution from 0 to 20, which should be sufficiently vague to allow enough variability.

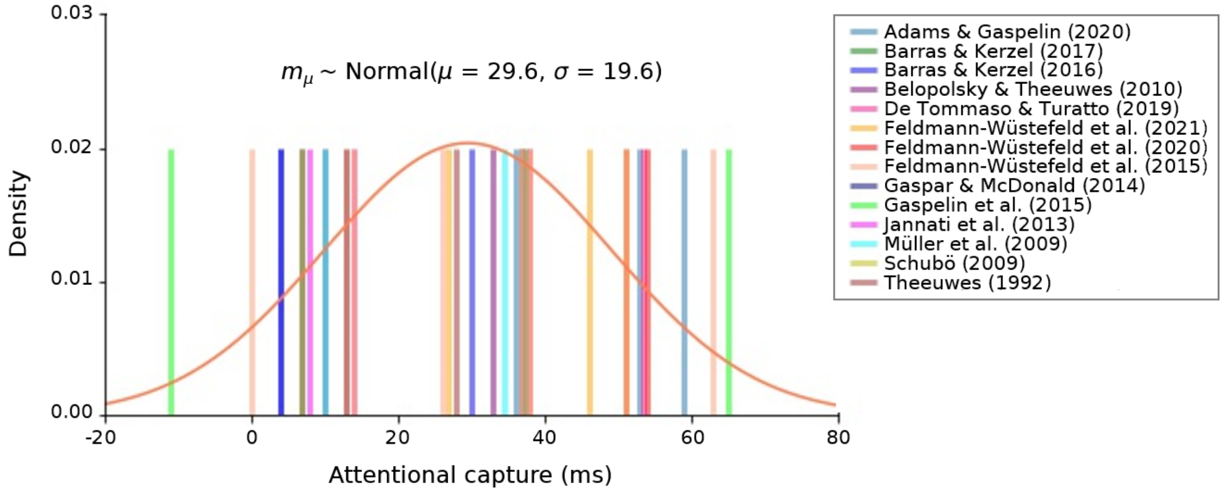


**Supplementary Figure S2.** A normal distribution based on attentional capture values from the literature (coloured lines). Values were obtained from 36 experiments from 14 studies. This distribution is used as a prior for parameter *m*.

## A4: Priors compared to the posteriors

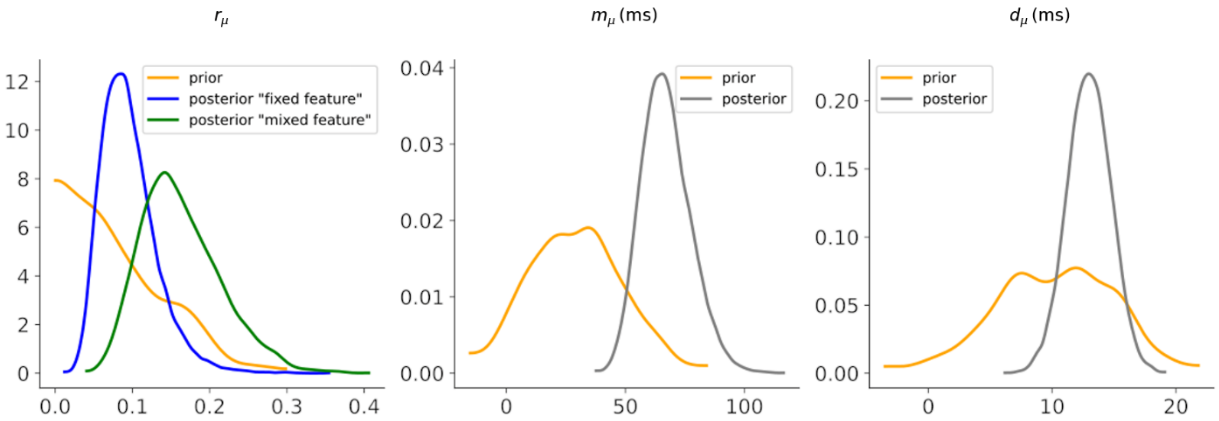


**Supplementary Figure S3.** Visualization of prior and posterior distributions. Shown are the prior predictive distributions (orange lines) and the posterior distributions for the fixed-feature task (blue line) and the mixed-feature task (green line). For the parameter $m_\mu$ and the parameter $d_\mu$ common group-level distributions were used (grey lines).

## A5: Posterior modes

| Parameters | Fixed-feature task | Mixed-feature task | Between tasks | Unit |
|---|---|---|---|---|
| $r_\mu$ | 0.08 [0.04, 0.17] | 0.14 [0.07, 0.27] | 0.06 [-0.02, 0.17] | |
| $m_\mu$ | Common group level: 64 [48, 87] | | - | ms |
| $d_\mu$ | Common group level: 13 [9.6, 16] | | - | ms |

**Supplementary Table S1.** Posterior modes of the parameter estimation. Parameter $r_\mu$ is the spatial gradient of suppression, parameter $m_\mu$ is the maximum capture at locations where suppression has faded out, and parameter $d_\mu$ is the residual capture at the high-probability location. Square brackets show 95% highest posterior density.

## A6: 3D visualization of the suppression map per task

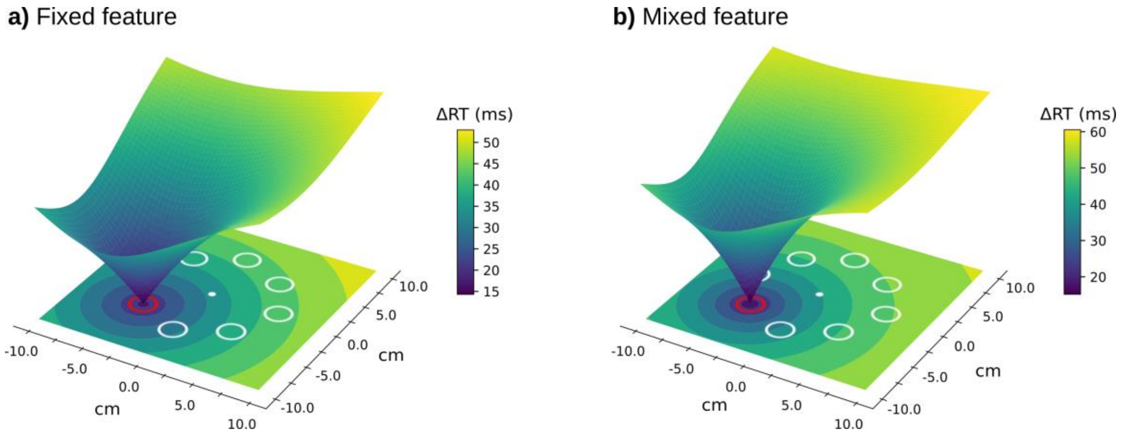


**Supplementary Figure S4.** 3D visualization of suppression map. (**a**) Fixed-feature task. (**b**) Mixed-feature task. The colours show the estimates of the spatial gradient of suppression. The high-probability location is indicated by the colour distractor.

## A7: Potential contribution to a priority map

If we interpret the suppression map as a factor that contributes to an overall priority map, the question arises how it interacts with, for instance, stimulus-driven activation. One possible interaction is illustrated in Figure S5.

Figure S5 (label a) shows how stimulus-driven and goal-driven activations combine to an integrated priority map. The strength of activations at each location on this map determines how attention is deployed.
In experiments like that of the present study, selecting the target or the distractor would be proportional to the ratio of the activations at the respective locations. In the beginning, the distractor activation is rather still strong, hence distractors capture attention frequently. In this example, the limited template precision leads to a low signal-to-noise ratio (Figure S5, label b). Hence, non-target and distractor locations receive some goal-driven activation, and consequently, in early trials, the distractor is a strong competitor to the target for selection. A more precise target template can lead to a more distinct target activation in the priority map, rendering capture by the distractor less likely. Whenever the distractor captures attention, a feedback signal is sent to the suppression map (Figure S5, label c), strengthening suppression of the respective location. This automatically means: less distractor capture (e.g., with more specific templates) leads to less frequent "deepening" of the suppression valley at the distractor location (i.e. less distractor location learning). Moreover, as the location gets more and more suppressed (Figure S5, label d), the capture occurs less often, causing the decline to be fast in the beginning and then level off, in agreement with our exponential learning curve model. Overall, this process reflects the transition from initially stimulus-driven toward predominantly goal-driven attentional guidance (see Vecera et al.[19]).

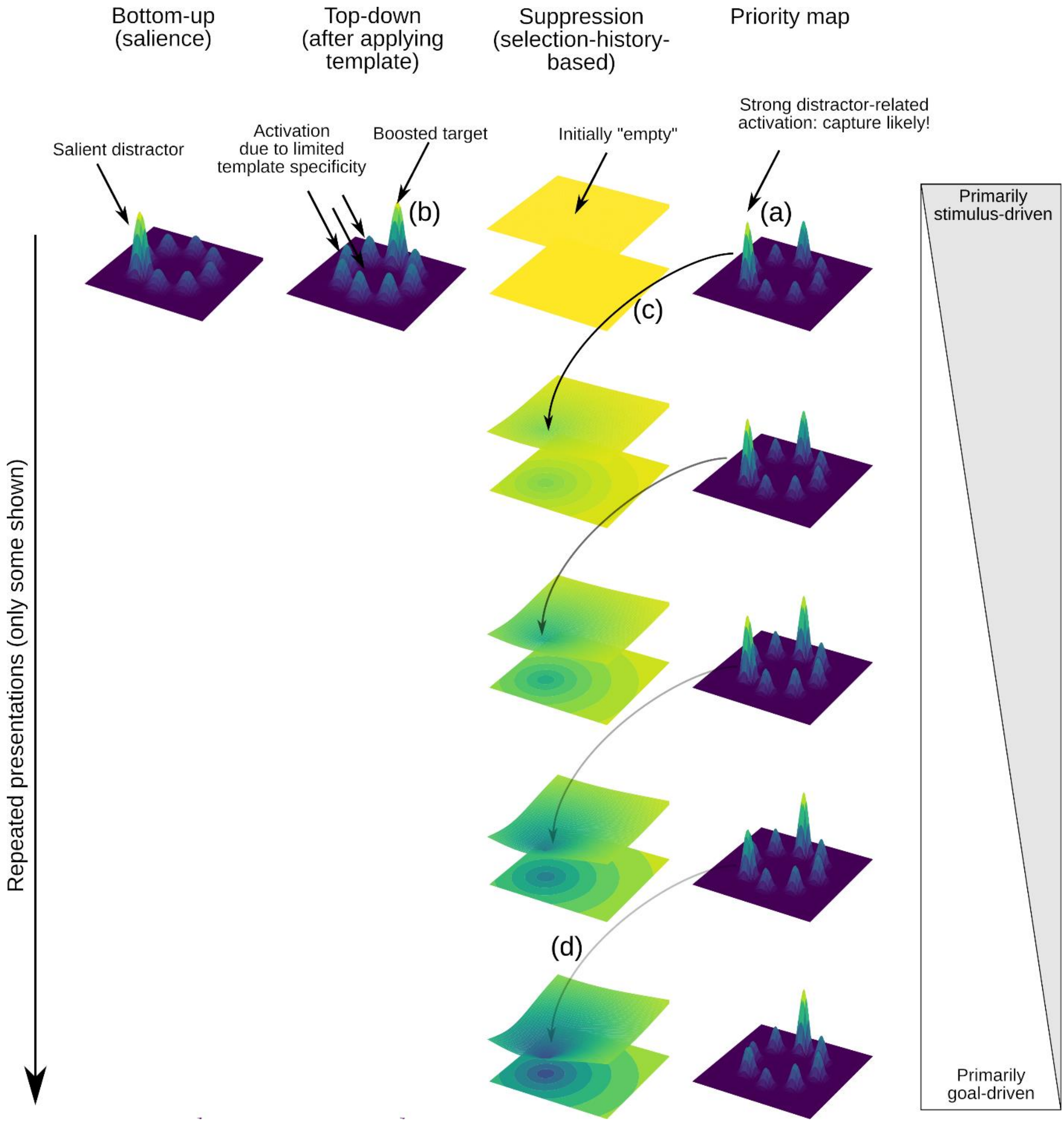


**Supplementary Figure S5**. In this hypothetical process, the priority map is a combination of stimulus-driven and goal-driven activations, and the suppression map. Stimulus-driven and goal-driven activations in this illustration would already be the combinations across feature dimensions[20]. The rows in this figure show some exemplary high-probability location trials. The labels **(a)** to **(d)** are explained in the text. The rightmost column of the figure was adopted from Vecera et al.[19].

## Supplementary Methods B: Learning curve model

The learning curve model describes how the RTs decrease over the course of the experiment and is described in the methods section of the paper.

*Note:* The code for the model implementation can be found at https://github.com/AylinH/HanneEtAl2022_DisLocLearning. Moreover, the traces sampled from the model are available at https://osf.io/5wcex/ and can be assessed using ArviZ[2] if more details are needed than presented here.

### B1: Model structure, priors, sampling parameters

Hyperpriors:
*Note:* Due to the exponential transformation on the participant-level, these hyperpriors effectively parameterize log-normal group-level distributions.

$$s_\mu^j \sim \text{Normal}(\mu = 1.8, \sigma = 1)$$

$$a_\mu^{(j)} \sim \text{Normal}(\mu = -0.5, \sigma = 0.5) \qquad a_\sigma^{(j)} \sim \text{HalfCauchy}(\beta = 0.5)$$

$$c_\mu^{(j)} \sim \text{Normal}(\mu = 1.8, \sigma = 1) \qquad c_\sigma^{(j)} \sim \text{HalfCauchy}(\beta = 0.5)$$

$$\text{sd}_\mu \sim \text{Normal}(\mu = -2, \sigma = 1) \qquad \text{sd}_\sigma \sim \text{HalfCauchy}(\beta = 0.1)$$

Parameters for each participant $i$

$$s_{z_i} \sim \text{Normal}(\mu = 0, \sigma = 1) \qquad s_i \leftarrow \exp\left(s_\mu + s_{z_i} \cdot \text{s}_\sigma\right)$$

$$\text{sd}_{z_i} \sim \text{Normal}(\mu = 0, \sigma = 1) \qquad \text{sd}_i \leftarrow \exp\left(\text{sd}_\mu + \text{sd}_{z_i} \cdot \text{sd}_\sigma\right)$$

Asymptote and rate parameters in variants without condition effects:

$$a_{\epsilon i} \sim \text{Normal}(\mu = 0, \sigma = 1) \qquad a_i \leftarrow \exp\left(a_\mu + a_{\epsilon_i} \cdot a_\sigma\right)$$

$$c_{\epsilon i} \sim \text{Normal}(\mu = 0, \sigma = 1) \qquad c_i \leftarrow \exp\left(c_\mu + c_{\epsilon_i} \cdot c_\sigma\right)$$

Superscript $j$ refers to baseline, high-, and low-probability conditions concerning $a$ and $c$. With respect to $s$, high- and low-probability are treated as one condition. See main text. Subscript $i$ refers to the individuals.

The parameters for different conditions are modelled (using LKJ Cholesky Covariance Priors) by multivariate Normal distributions over the conditions and participants. This establishes correlated varying effects that take into account that the data from the same participant in different conditions is correlated.

$$s_{z_i}^{\ j} \sim \text{Normal}(\mu = 0, \sigma = 1)$$

$$s_\text{col}, s_\sigma{}^j \sim \text{LKJCholeskyCov}(2, \eta = 1,\ \text{sd\_dist} = \text{HalfCauchy}(\beta = 1))$$

$$s_i^j \leftarrow \exp\left(s_\mu^j + s_\text{col} \cdot s_{z_i}^{\ j}\right)$$

$$a_{z_i}^{\ j} \sim \text{Normal}(\mu = 0, \sigma = 1)$$

$$a_\text{col}, a_\sigma{}^j \sim \text{LKJCholeskyCov}(3, \eta = 1,\ \text{sd\_dist} = \text{HalfCauchy}(\beta = 1))$$

$$a_i^j \leftarrow \exp\left(a_\mu^j + a_\text{col} \cdot a_{z_i}^{\ j}\right)$$

$$c_{z_i}^{\ j} \sim \text{Normal}(\mu = 0, \sigma = 1)$$

$c_{\text{col}}, {c_\sigma}^j \quad \sim \quad \text{LKJCholeskyCov}(3, \eta = 1,\ \text{sd\_dist} = \text{HalfCauchy}(\beta = 1))$

$c_i^j \quad \leftarrow \quad \exp\left(c_\mu^j + c_{\text{col}} \cdot {c_z}_i^j\right)$

Transformations on the group level

$s_{\text{group mean}}^j \quad \leftarrow \quad \exp\left(s_\mu^j + {s_\sigma^j}^2/2\right)$

$a_{\text{group mean}}^j \quad \leftarrow \quad \exp\left(a_\mu^j + {a_\sigma^j}^2/2\right)$

$c_{\text{group mean}}^j \quad \leftarrow \quad \exp\left(c_\mu^j + {c_\sigma^j}^2/2\right)$

Observations:

$\text{RT}_i^j(x) \quad \sim \quad \text{Normal}(\mu = a_i^j + \left(s_i^j - a_i^j\right)(x+1)^{-c_i^j}, \sigma = sd_i)$

MCMC Sampling parameters (PyMC[3]):

| | |
|---|---|
| Sampler: | NUTS[4] |
| Number of posterior predictive samples: | 20,000 (5,000 after thinning) |
| Number of iterations to tune: | 1,000 |
| Target acceptance rate: | 0.90 |
| Initialization method used: | adapt_diag |

## B2: Priors compared to the posteriors

As it is difficult to grasp how informative the log-normal hyperpriors are, we present visualizations of the prior predictive and the posterior distributions for the $s^{j}_{\text{group mean}}$, $a^{j}_{\text{group mean}}$, $c^{j}_{\text{group mean}}$ which are on the data scale. The prior predictive distributions are all relatively flat compared to the posterior distributions and only vaguely informative, showing that the data drove the estimates.

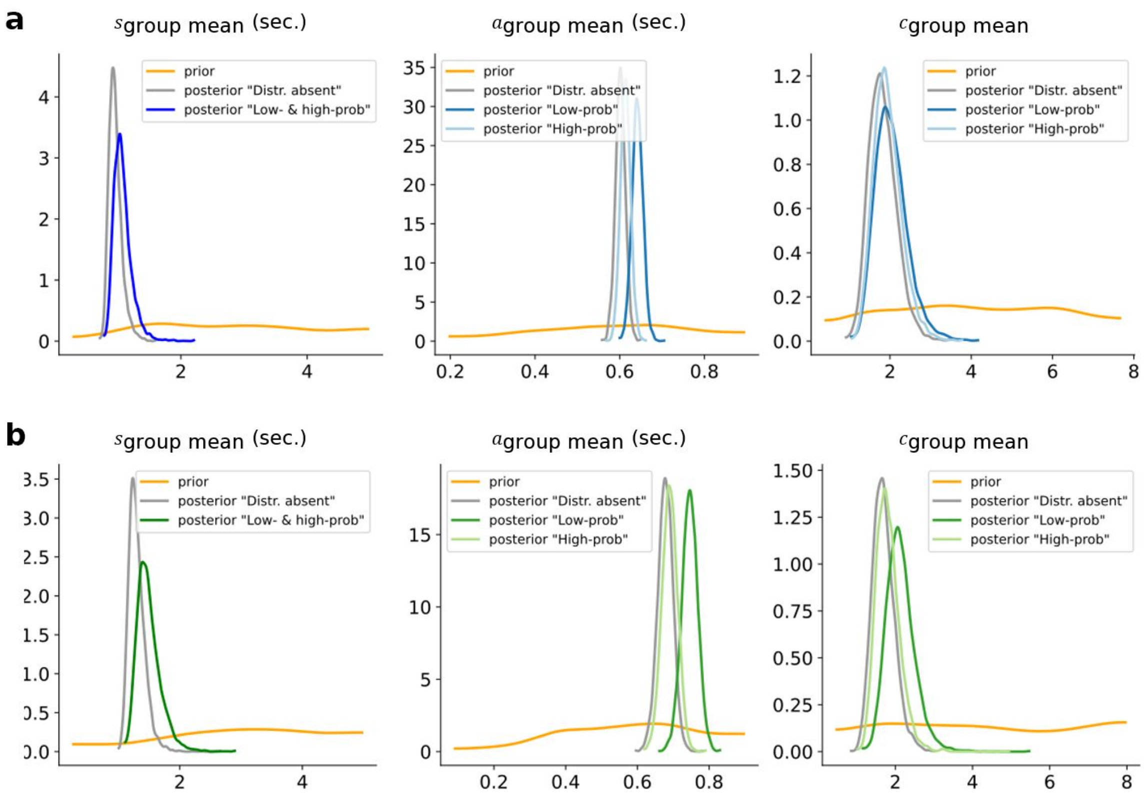


**Supplementary Figure S6.** Visualization of the priors and posteriors. **(a)** Fixed-feature task: prior predictive distributions (orange lines) and posterior distributions are shown for the starting RT level (black line) and the distractor conditions high-prob. (light blue line), low-prob. (dark blue line) and distractor absent (grey line). **(b)** Mixed-feature task: prior predictive distributions (orange lines) and posterior distributions are shown for the starting RT level (black line) and the distractor conditions high-prob. (light green line), low-prob. (dark green line) and distractor absent (grey line). The posteriors are computed based on the both-effects-model, which allows for effects on the asymptote and on the decrease rate parameter.

## B3: Model comparison

| Model | Rank | Loo | pLOO | dLOO | dSE | Weight |
|---|---|---|---|---|---|---|
| ***Fixed-feature task*** | | | | | | |
| Asymptotic RT level effect | 0 | 7298.95 | 171.40 | 0.00 | 0.00 | 0.83 |
| Both effects | 1 | 7287.83 | 188.83 | 11.12 | 2.73 | 0.00 |
| Decrease rate effect | 2 | 7275.56 | 170.14 | 23.39 | 6.76 | 0.00 |
| No effect | 3 | 7264.05 | 146.99 | 34.90 | 10.51 | 0.17 |
| ***Mixed-feature task*** | | | | | | |
| Asymptotic RT level effect | 0 | 3494.70 | 130.04 | 0.00 | 0.00 | 0.94 |
| Both effects | 1 | 3480.40 | 146.22 | 14.30 | 3.88 | 0.00 |
| Decrease rate effect | 2 | 3452.83 | 131.97 | 41.87 | 6.02 | 0.00 |
| No effect | 3 | 3451.41 | 109.54 | 43.29 | 10.08 | 0.06 |

**Supplementary Table S2.** Model comparison for both search tasks. Rank indicates the ranking of the model in the comparison, starting with the best model (rank 0) to the worst model (rank 3) based on its leave-one-out cross-validation (Loo) score. pLOO is the estimated effective number of parameters, dLOO indicates the relative difference of the LOO score to the best model, dSE indicates its standard error, weight estimates the model's weight given if it were used in model averaging.

## B4: Participant-level estimates

Search strategies are determined by various factors (e.g., Adam et al.[21]; Irons & Leber[22]), and some participants tend to use search strategies that are, from an optimal observer's point of view, not very efficient. Accordingly, some participants might have searched for a shape singleton in our fixed-feature search task rather than searching for the exact target feature. Leber and Egeth[23] found that participants stick with the search mode they experienced as being effective in previous search trials. For instance, participants who had performed singleton search for several trials continued to search for a singleton target even though the search arrays allowed searching for a specific target feature (see also Bacon & Egeth[24]).

We looked at the participant-level estimates for each individual's learning curve parameters to examine whether there is a mix of searchers who apply feature search and searchers who apply singleton detection in the fixed-feature task. To look for evidence for subgroups with different strategies, we took several measures: We plotted the individual means for distractor-absent and present trials in the fixed-feature task to check for possible differences in the data patterns. Participants that use singleton search should show slower RTs and, as they are vulnerable to any singleton, larger capture effects when distractors were presented at low-probable locations. No such pattern was visible in the data (see Figure S7a). Also, if there was a mixture of search modes, we assume that the variability is greater in the fixed-feature task than in the mixed-feature task. However, the standard deviation is larger in the mixed-feature task (SD = 57.3) than in the fixed-feature task (SD = 45.56).

Concerning our modeling results, with a mixture of search modes, we would also expect larger variability in the fixed-feature task's starting RT level, or if the different strategies only manifest over time, in the asymptotic RT levels. We plotted the RTs of the starting level in distractor-absent trials (see Figure S7b) and the asymptotic RTs (see Figure S7c) for each participant. If different search modes were used in the fixed-feature task, we would expect to see the estimated values scatter around two subgroups. One subgroup would be similarly slow as the participants in the mixed-singleton task and the other subgroup would tend to be faster. We see no such pattern (or any other conspicuity) in the starting level or asymptotic RTs. The

participants in the fixed-feature task seem to come from one group that tends to be faster than the mixed-feature group.

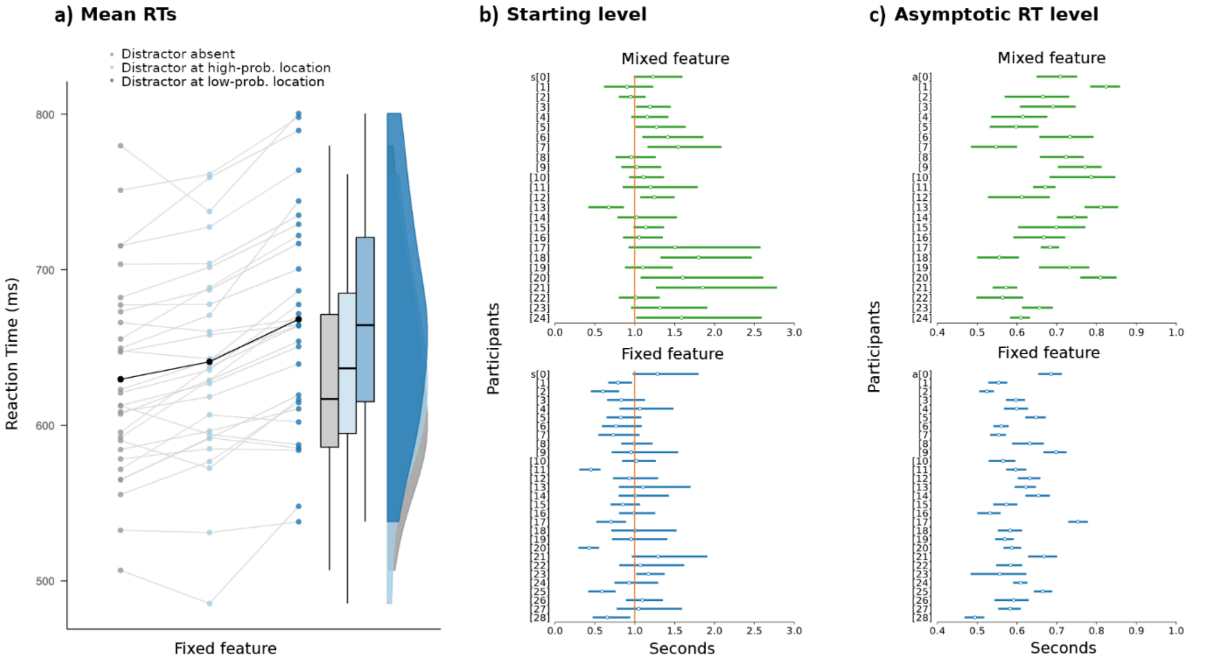


**Supplementary Figure S7. a**) Search response times for the fixed-feature task. Box-whisker plots show mean response times as a function of distractor-absent trials (grey), and distractor-present trials (coloured), with the distractor presented at the high-probability location (light blue) and at the low-probability locations (dark blue). **b**) Forest plots showing the *starting RT level* for each participant with the mode (circles), HPD (horizontal lines) for the fixed-feature task (blue, lower panel) and the mixed-feature task (green, upper panel). **c**) Forest plots showing the *asymptotic RT levels* for each participant; the logic of visualization is the same as in b). Best viewed digitally with magnification.

## Supplementary References


1. Kruschke, J. K. & Liddell, T. M. Bayesian data analysis for newcomers. *Psychon. Bull. Rev.* **25**, 155–177 (2018).
2. Kumar, R., Carroll, C., Hartikainen, A. & Martin, O. ArviZ a unified library for exploratory analysis of bayesian models in python. *J. Open Source Softw.* **4**, 1143 (2019).
3. Salvatier, J., Wiecki, T. V. & Fonnesbeck, C. Probabilistic programming in python using pymc3. *PeerJ Comput. Sci.* **2**, e55 (2016).
4. Hoffman, M. D. & Gelman, A. The no-U-turn sampler: adaptively setting path lengths in hamiltonian monte carlo. *J. Mach. Learn. Res.* **15**, 1593–1623 (2014).
5. Adams, O. J. & Gaspelin, N. Assessing introspective awareness of attention capture. *Attention, Perception, Psychophys.* **82**, 1586–1598 (2020).
6. Barras, C. & Kerzel, D. Salient-but-irrelevant stimuli cause attentional capture in difficult, but attentional suppression in easy visual search. *Psychophysiology* **54**, 1826–1838 (2017).
7. Jannati, A., Gaspar, J. M. & McDonald, J. J. Tracking target and distractor processing in fixed-feature visual search: Evidence from human electrophysiology. *J. Exp. Psychol. Hum. Percept. Perform.* **39**, 1713–1730 (2013).
8. Müller, H. J., Geyer, T., Zehetleitner, M. & Krummenacher, J. Attentional capture by salient color singleton distractors is modulated by top-down dimensional set. *J. Exp. Psychol. Hum. Percept. Perform.* **35**, 1–16 (2009).
9. Theeuwes, J. Perceptual selectivity for color and form. *Percept. Psychophys.* **51**, 599–606 (1992).
10. Schubö, A. Salience detection and attentional capture. *Psychol. Res.* **73**, 233–243 (2009).
11. Barras, C. & Kerzel, D. Active suppression of salient-but-irrelevant stimuli does not underlie resistance to visual interference. *Biol. Psychol.* **121**, 74–83 (2016).
12. Belopolsky, A. V. & Theeuwes, J. No capture outside the attentional window. *Vision Res.* **50**, 2543–2550 (2010).
13. De Tommaso, M. & Turatto, M. Learning to ignore salient distractors: Attentional set and habituation. *Vis. cogn.* **27**, 214–226 (2019).
14. Feldmann-Wüstefeld, T., Weinberger, M. & Awh, E. Spatially guided distractor suppression during visual search. *J. Neurosci.* **41**, 3180–3191 (2021).
15. Feldmann-Wüstefeld, T., Busch, N. A. & Schubö, A. Failed suppression of salient stimuli precedes behavioral errors. *J. Cogn. Neurosci.* **32**, 367–377 (2019).
16. Feldmann-Wüstefeld, T., Uengoer, M. & Schubö, A. You see what you have learned. Evidence for an interrelation of associative learning and visual selective attention. *Psychophysiology* **52**, 1483–1497 (2015).
17. Gaspar, J. M. & McDonald, J. J. Suppression of salient objects prevents distraction in visual search. *J. Neurosci.* **34**, 5658–5666 (2014).
18. Gaspelin, N., Leonard, C. J. & Luck, S. J. Direct evidence for active suppression of salient-but-irrelevant sensory inputs. *Psychol. Sci.* **26**, 1740–1750 (2015).

19. Vecera, S. P., Cosman, J. D., Vatterott, D. B. & Roper, Z. J. J. *The control of visual attention. Toward a unified account*. *Psychology of Learning and Motivation - Advances in Research and Theory* **60**, (Elsevier Inc., 2014).

20. Liesefeld, H. R. & Müller, H. J. Distractor handling via dimension weighting. *Curr. Opin. Psychol.* **29**, 160–167 (2019).

21. Adam, K. C. S., Patel, T., Rangan, N. & Serences, J. T. Classic visual search effects in an additional singleton task: An open dataset. *J. Cogn.* **4**, 1–10 (2021).

22. Irons, J. L. & Leber, A. B. Developing an individual profile of attentional control strategy. *Curr. Dir. Psychol. Sci.* **29**, 364–371 (2020).

23. Leber, A. B. & Egeth, H. E. Attention on autopilot: Past experience and attentional set. *Vis. cogn.* **14**, 565–583 (2006).

24. Bacon, W. F. & Egeth, H. E. Overriding stimulus-driven attentional capture. *Percept. Psychophys.* **55**, 485–496 (1994).
